# Using Machine Learning and Optical Microscopy Image Analysis of Immunosensors Made on Plasmonic Substrates: Application to Detect the SARS-CoV-2 Virus

**DOI:** 10.1021/acssensors.4c03451

**Published:** 2025-02-17

**Authors:** Pedro R. A. Oiticica, Monara K. S. C. Angelim, Juliana C. Soares, Andrey C. Soares, José L. Proença-Módena, Odemir M. Bruno, Osvaldo N. Oliveira

**Affiliations:** 1 São Carlos Institute of Physics (IFSC), University of São Paulo (USP), São Carlos, SP 13566-590, Brazil; 2 Nanotechnology National Laboratory for Agriculture (LNNA), Embrapa Instrumentação, São Carlos, SP 13560-970, Brazil; 3 Department of Genetics, Evolution, Microbiology and Immunology, Institute of Biology, University of Campinas, Campinas, SP 13083-862, Brazil; 4 Experimental Medicine Research Cluster (EMRC), University of Campinas, Campinas, SP 13083-862, Brazil

**Keywords:** plasmonic substrates, immunosensor, SARS-CoV-2 virus, computer vision, machine learning

## Abstract

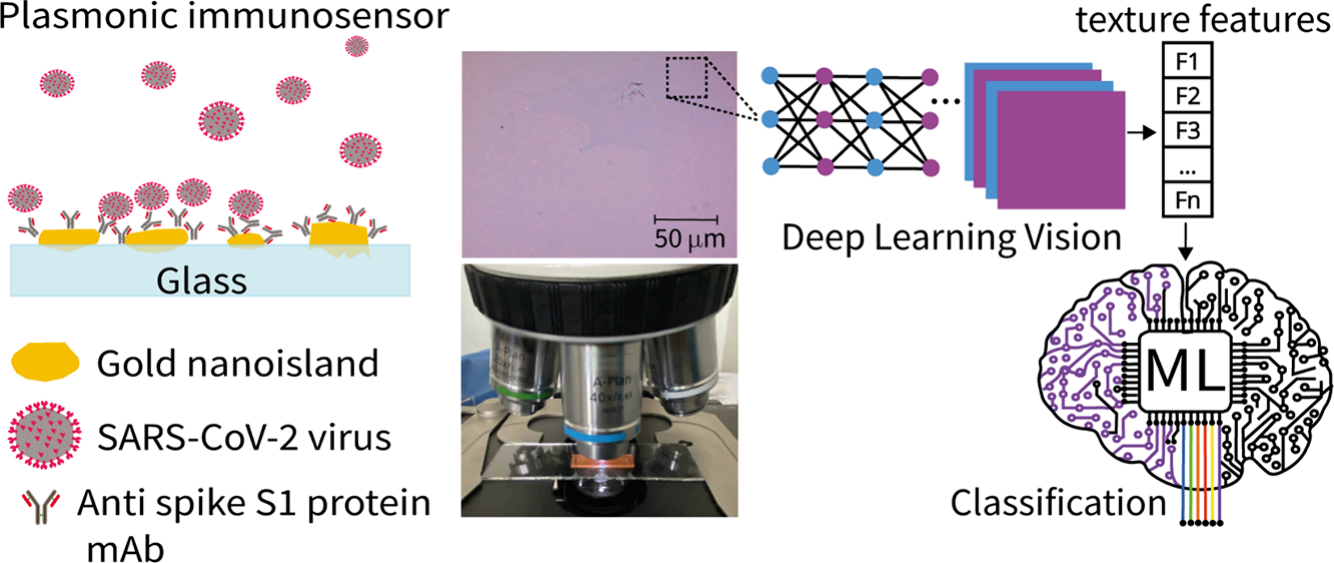

In this article, we introduce a diagnostic platform comprising an optical microscopy image analysis system coupled with machine learning. Its efficacy is demonstrated in detecting SARS-CoV-2 virus particles at concentrations as low as 1 PFU (plaque-forming unit) per milliliter by processing images from an immunosensor on a plasmonic substrate. This high performance was achieved by classifying images with the support vector machine (SVM) algorithm and the MobileNetV3_small convolutional neural network (CNN) model, which attained an accuracy of 91.6% and a specificity denoted by an F1 score of 96.9% for the negative class. Notably, this approach enabled the detection of SARS-CoV-2 concentrations 1000 times lower than the limit of detection achieved with localized surface plasmon resonance (LSPR) sensing using the same immunosensors. It is also significant that a binary classification between control and positive classes using the MobileNetV3_small model and the random forest algorithm achieved an accuracy of 96.5% for SARS-CoV-2 concentrations down to 1 PFU/mL. At such low concentrations, straightforward screening of newly infected patients may be feasible. In supporting experiments, we verified that texture was the main contributor to the distinguishability of images taken at different SARS-CoV-2 concentrations, indicating that the combination of ML and image analysis may be applied to any biosensor whose detection mechanism is based on adsorption.

The need for rapid, reliable, and cost-effective diagnostic tools to detect viral infections has been highlighted by the COVID-19 pandemic. Various biosensing platforms, including those based on plasmonic nanomaterials, have been developed to detect the SARS-CoV-2 virus. These plasmonic platforms exploit the unique optical properties of nanostructures to detect viral particles with high sensitivity through localized surface plasmon resonance (LSPR).^1−5^ The sensitivity of these sensors depends on the shift in LSPR spectra upon binding target molecules to functionalized nanostructures.^6^ Since each type of plasmonic structure has a specific LSPR band, the analysis process is specific to each nanostructure. In addition, the response of the LSPR spectrum is different at each point; i.e., the maximum peak of the band does not always provide the most sensitive response.^7^ While LSPR-based detection offers high sensitivity, it requires expensive spectrometers, limiting its widespread adoption in clinical settings^8^ or in point-of-care (PoC) devices. Other techniques for optical detection in plasmonic biosensors, such as surface-enhanced Raman scattering (SERS),^9,10^ fluorescence microscopy,^11^ and ellipsometry,^12^ use sophisticated equipment that is rarely available in hospitals.

Highly sensitive immunosensors have been proposed for the SARS-CoV-2 virus with diverse detection methods, including amperometry, electrochemical and electrical impedance spectroscopies, colorimetry, and the use of electrical measurements with field effect transistors (FETs). These sensors had limit of detection (LOD) values on the order of 10^0^ to 10^1^ PFU/mL.^13−17^ It is not straightforward to determine the number of virus particles in clinical samples that would correlate with these limits of detection because this number depends on the type of body fluid and the stage of the infection. Samples from infected patients collected from the respiratory tract, nasopharynx, and saliva can contain virus particles corresponding to the range of 10^1^ to 10^6^ plaque-forming units per mL (PFU/mL).^18^

An alternative approach involves optical microscopy, which is available in laboratories and hospitals and offers a more affordable solution compared to spectroscopies. Image processing of plasmonic biosensors has been made mostly with handcrafted algorithms that consider only statistical measurements of the RGB (“red–green–blue”) intensities.^4,11,19^ Detection of the SARS-CoV-2 virus using plasmonic sensor imaging, for example, was demonstrated by Liang et al.^4^ using substrates formed by gold nanocups. The image analyses selected color characteristics of the RGB histograms and the values of hue of the images converted from the RGB space to the HSV (hue, saturation, and value) space. The binary classification was made with the support vector machine (SVM) method, obtaining 97% accuracy. Only the average color variation information was used to train the classification model, without considering texture and spatial information on the image. Since efficient computer vision algorithms exist for feature extraction in images, both based on convolutional neural networks (CNNs) and handcrafted extraction algorithms,^20−23^ we believe that they could be combined with machine learning (ML) to automate diagnosis procedures.

In this paper, we demonstrate that ML and image analysis of plasmonic biosensors can yield a high performance, even higher than that using LSPR. This will be shown with a plasmonic immunosensor formed on gold nanoislands to detect SARS-CoV-2 virus particles.

## Materials and Methods

B270 glass slides, 1.0–1.2 mm thick, were acquired from Schott. The antibodies against anti-SARS-CoV-2 Spike glycoprotein S1 mAb [CR3022] (ab273073) were purchased from ABCAM (USA). 11-MUA (11-mercaptoundecanoic acid), EDC (*N*-(3-dimethilaminopropyl)-*N*′-ethylcarbodiimide hydrochloride), and NHS (*N*-hydroxysuccinimide) were obtained from Sigma-Aldrich (USA). Washing procedures employed isopropanol 99.5% (Synth, Brazil) and ethanol 99.8% (Exodo Cientifica, Brazil), while aqueous solutions were obtained from a Milli-Q water purification system with 18.2 MΩ·cm resistivity (Millipore Integral 10). The phosphate buffer saline (PBS)/MgCl_2_ buffer was prepared with NaCl 137 × 10^–3^ mol L^–1^, Na_2_HPO_4_ 10 × 10^–3^ mol L^–1^, KH_2_PO_4_ 1.7 × 10^–3^ mol L^–1^, and KCl 2.7 × 10^–3^ mol L^–1^, adjusted to pH 7.4 and added with MgCl_2_ 1.0 × 10^–3^ mol L^–1^. The SARS-CoV-2 B.1 strain (HIAE-02-SARS-CoV-2/SP02/human/2020/BRA; GenBank MT126808.1) was isolated from Brazil’s second confirmed COVID-19 case, and the respiratory syncytial virus was from subgroup A (RSV A2 strain). For viruses’ stock preparation, Vero cells (ATCC CCL81) were infected at a multiplicity of infection (MOI) of 0.1 for 1 h with gentle agitation at 15 rpm. After this adsorption phase, the cells were washed with prewarmed PBS, cultured in DMEM with 10% heat-inactivated fetal bovine serum and 1% penicillin–streptomycin, and incubated at 37 °C in 5% CO_2_. The supernatant was collected 2 to 3 days postinfection and stored at −80 °C. Virus inactivation was achieved through ultraviolet (UV) irradiation under biosafety conditions following Patterson et al.^24^ The titration of the virus was determined using plaque-forming unit assays. The viruses’ strains were sourced from the Laboratory for the Study of Emerging Viruses (LEVE) at the Institute of Biology, UNICAMP, Brazil.

### Immunosensor Fabrication on AuNI/Glass Plasmonic Substrates

Glass slides with dimensions of 25 × 8 × 1.0 mm were cleaned in an ultrasonic thermal bath at 65 °C for 20 min in neutral detergent solution Extran MA02 (from Merck Supelco) diluted with a ratio of 1:10 v/v ultrapure water for 10 min and isopropanol (99.5% Synth, Brazil) for 10 min. The substrates were then treated with UV/ozone for 10 min, rinsed in Milli-Q ultrapure water, and dried under a nitrogen flow. A 6 nm thick gold film (Au/glass) was deposited with the MB-Evap evaporator inside a LabMaster 130 Glovebox (MBraun) at a chamber pressure of 1 × 10^–6^ mbar at a film growth rate of 0.03 nm/s. The speed and thickness of the gold film were controlled during deposition by using a quartz crystal microbalance (QCM) inside the evaporation chamber. The Au/glass films were annealed inside muffle furnace model EDGCON 5P (EDG, Brazil) at 600 °C for 2 h. This method was adapted from Tesler et al.^25^ After thermal annealing, the resulting plasmonic AuNI/glass substrates were cleaned with ultrasonic thermal bath in isopropanol for 10 min and ultrapure water for 10 min, dried under a nitrogen flow, and sterilized in UV/ozone for 10 min. The final cleaning processes removed the remaining dust particles from the manufacturing processes and loosely adhered AuNIs from the glass surface. They also sterilized the plasmonic surface for biosensing. The morphology of the resulting AuNI/glass substrates are shown in Figure S1 of the Supporting Information. We formed an 11-MUA self-assembled monolayer (SAM) on the AuNIs' surface by incubation of the plasmonic substrates in 11-MUA/ethanol 10 mM solution for 24 h at room temperature (25 °C). The SAM-coated substrates were rinsed in pure ethanol and dried under a N_2_ flow. We activated the carboxylic acid (−COOH) terminals using the EDC/NHS reaction, immersing the substrates in a solution of 0.1 mol L^–1^ EDC and 0.1 mol L^–1^ NHS with equal volumes, followed by a 30 min incubation at room temperature. Then, the substrates were immersed in ultrapure deionized water and dried under a N_2_ flow. The anti-SARS-CoV-2 mAb antibody corresponding to the Spike S1 protein of the SARS-CoV-2 virus was immobilized by dropping a 0.1 mg/mL solution in PBS/MgCl_2_ followed by a 2 h incubation at room temperature.

### Detection Procedures with the Plasmonic Immunosensor

The immunosensor was immersed in a tube containing 1 mL of the test solution for 30 min at room temperature (25 °C). Next, the sensor was rinsed in a PBS/MgCl_2_ buffer solution and dried using a N_2_ stream. We performed LSPR measurements before and after the tests in the same region of the immunosensor. Optical microscopy images were acquired before and after the tests. We characterized the LSPR spectrum using the UV–vis fiber optic spectrometer (400–1000 nm) model USB4000 (Ocean Optics) with the tungsten halogen light source model LS-1 (Ocean Optics). The light source was collimated into an approximately 3 mm diameter beam, according to the scheme in Figure S2a in the Supporting Information. We used a sample holder with a translational stage to acquire the LSPR spectrum at the same region of the sensor before and after the tests. The spectra were analyzed with programs in Python to extract 10 features from the LSPR band: Peak_λ, Peak_abs, FWHM_λ, FWHM_abs, inf1_λ, inf1_abs, inf2_λ, inf2_abs, valley_λ, and valley_abs. Here, fwhm is the full width at half-maximum. The feature peak refers to the maximum absorbance of the LSPR band. Features in parts inf1 and inf2 correspond to the inflection points on the left and right sides of the LSPR band, respectively. The details about the features are given in Figure S4 in the Supporting Information. A single variable analysis was performed with plots for the features peak_λ and peak_abs. The multidimensional information in the 10 features extracted from the LSPR spectra was analyzed using the dimension reduction and information visualization method Interactive Document Mapping (IDMAP)^26,27^ with the software PEx-Sensors (details of the IDMAP method are included in the Supporting Information).

### Optical Microscopy, Computer Vision, and Machine Learning Methods

Optical microscopy images were obtained with the Zeiss Axio Lab.A1 transmission optical microscope (Carl Zeiss) with the Zeiss A-Plan 40×/0.65 objective, C-mount adapter with 0.63× magnification, and CMOS sensor model AxioCam ERc 5s. The images have 1920 × 2560 pixels corresponding to a field of view of approximately 165 × 220 μm. The total magnification is equivalent to that observed in the eyepiece (400× magnification). Various computer vision and machine learning (ML) algorithms were compared for the classification of optical microscopy images of the plasmonic immunosensor. Computer vision methods to extract features take images as inputs and return a feature vector (also called an image descriptor) for each image. We employed five handcrafted methods*:* LBP (Local Binary Pattern),^28^ CLBP (Complete Local Binary Pattern),^29^ GLCM (Gray Level Co-occurrence Matrix),^30^ GLDM (Gray Level Difference Method)^31^ and RGB5D-LBP (RGB to 5D Local Binary Pattern). These algorithms (with the exception of RGB5D-LBP) are only applied to grayscale images, so we need to convert the images before applying these feature extractors. RGB5D-LBP was developed in this work based on the algorithm MCLBP proposed by Shu et al.^32^ by combining the classic LBP components of the three RGB channels and two perpendicular LBP components in a square predefined pattern coordinate. This feature extractor was conceptualized to be applied on three-channel images. Further details on the handcrafted methods are given in the Supporting Information.

We also employed 12 feature extraction methods based on deep learning models and convolutional neural networks (CNN): DenseNet121,^33^ EfficientNetV2_B0, EfficientNetV2_B1, EfficientNetV2_M, EfficientNetV2_S,^34^ MobileNet,^35^ MobileNetV2,^36^ MobileNetV3 small,^37^ ResNet18, ResNet34,^38^ VGG16, and VGG19.^39^ The network parameters of these architectures were imported (pretrained models) from the corresponding models trained with the ImageNet^40^ database. These CNN architectures were configured for feature extraction by removing the classification layer and adding the “Global Average Pooling” after the last convolutional tensor layer. CNN-based extractors can be applied to RGB or grayscale images. The parameters of the CNN layers were not adjusted during the training of the ML algorithms. These CNN architectures were chosen based on the GPU memory requirement, processing times, and the dimensionality of the descriptors being up to 1280. Further details about the CNN architectures used are given in the Supporting Information.

The image descriptors were used in training four machine learning models: LDA (linear discriminant analysis),^41^ KNN (K-nearest neighbors), SVM (support vector machines), and RF (random forest).^42^ LDA was applied with the least-squares solution solver (lsqr) and shrinkage using the Ledoit–Wolf lemma.^43^ In KNN, we set *K* = 5, and the SVM uses a linear kernel. The parameter of the RF was 200 trees, max_features = “sqrt” (in each search for the best split, it considers a number of features equal to the square root of the total number of features), and the other parameters were kept as the default by using scikit-learn implementation, version 1.4.1.

**Figure 1:**
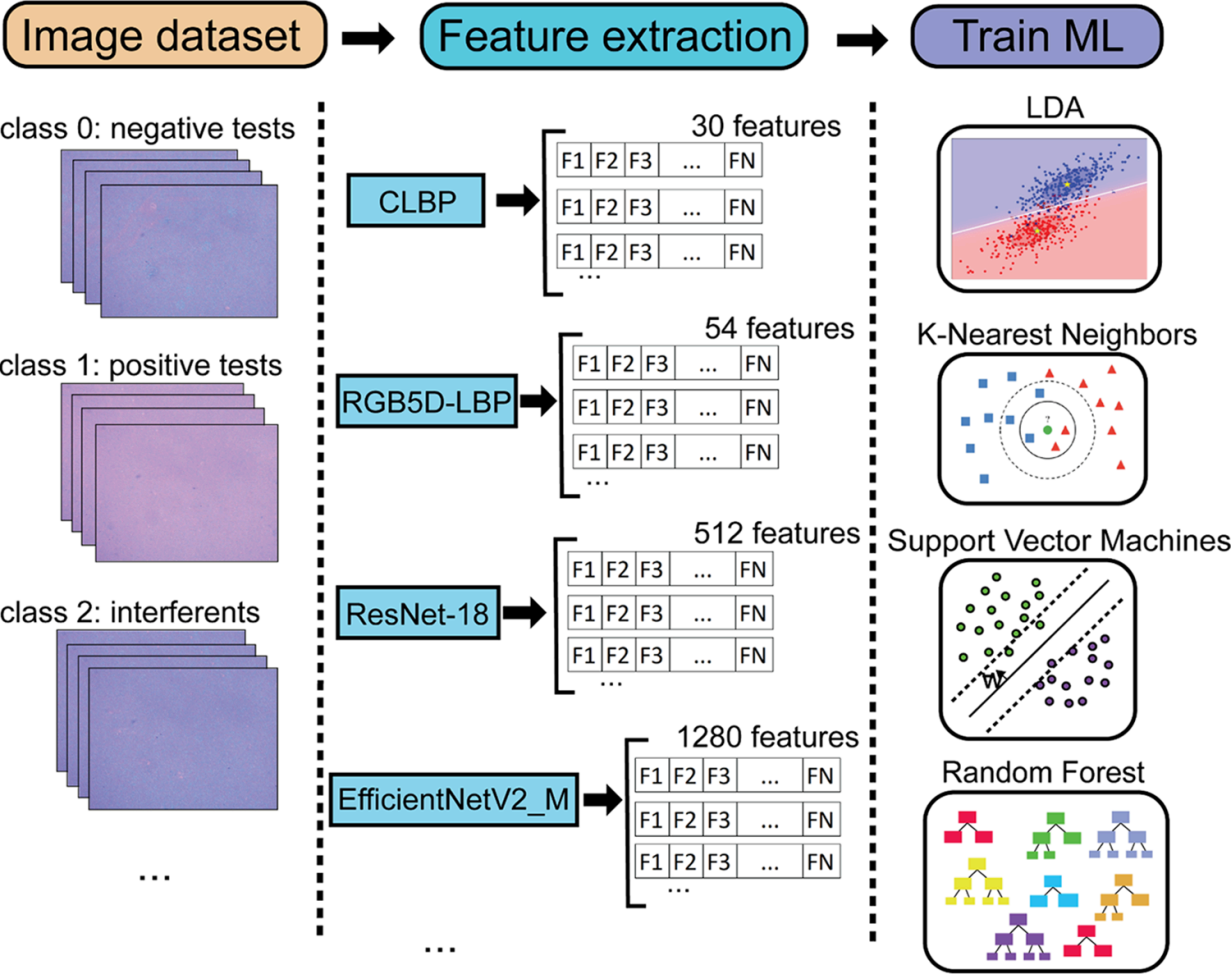
Diagram of image classification processes (“pipeline”) using computer vision and machine learning techniques.

The pipeline of image classification for the plasmonic immunosensor is shown in Figure 1. A brief description of the ML classifiers algorithms is found in the Supporting Information. A set of images is taken and organized into different classes. We apply computer vision methods to extract features of the images; then for each method, we obtain a set of feature vectors. The feature vector set corresponding to each computer vision method is used to train the machine learning models LDA, KNN, SVM, and RF. To evaluate each model, we performed stratified fivefold repeated three times cross-validation, measuring the following metrics: accuracy, recall, precision, F1 score, negative predictive value, and true negative rate of the test sets. For unbalanced data sets, it is recommended to perform stratification in each cross-validation iteration to ensure that the number of samples in each class maintains the same proportion as in the original data set. The details about the stratified k-fold process are included in the Supporting Information, which also contains the equations of the metrics considered.

We employed IDMAP to project the *n*-dimensional predicted probabilities (where *n* is the number of classes considered) for each sample predicted by the ML classifiers. This method of information visualization creates a 2D map by conserving the dissimilarity of the points in the predicted probabilities space. In this analysis, the ML algorithm was trained with the entire data set, and then we applied the trained algorithm to perform predictions for all samples in the data set. The predicted probabilities were then applied to the IDMAP method. The objective of this analysis is to visualize how the ML algorithm classifies each sample, the separation of the clusters of well-classified samples (higher concentrations), and the confusion in the classification of samples corresponding to tests in the lower-concentration dilutions. It is also possible to visualize outliers. Since this analysis is aimed at ML explainability and visualization of the prediction information for all samples in the data set, we did not separate the data in training and validation sets.

The handcrafted extractors were executed using programs in Python version 3.9 with Scikit-image library v. 0.20.^44^ The methods based on deep learning and CNN were implemented with programs in Python v. 3.9 using the libraries Keras v. 2.10.0, TensorFlow v. 2.10.1, and PyTorch v. 2.0.0 (pytorch-cuda v. 11.7). The ML models were implemented using the library Scikit-learn v. 1.4.1.^45^ The handcrafted feature extractors were executed in a laptop with processor Intel core i7-6700HQ CPU with four 2.6 GHz physical cores and 16 GB of RAM memory. The CNN feature extractors require a larger amount of dedicated memory, especially in the case of very large images (1920 × 2560 pixels and three channels). We ran the CNN feature extractors using the Google Colabcloud processing platform,^46^ which offers free use of a Jupyter notebook running Python language v. 3.10.12. The computational resources comprised an Intel Xeon CPU with two cores of @2.20 GHz, 13 GB RAM, 78.2 GB disk space, and an NVIDIA Tesla T4 GPU with 16 GB dedicated memory (VRAM).

## Results and Discussion

The main aim in this study is to demonstrate that diagnosis can be made by combining image analysis and machine learning, in which optical microscopy images are taken from the immunosensors after they are exposed to the samples under analysis. Since we used immunosensors that can also be used for detection using LSPR spectroscopy, we first present the results from LSPR, which will then be compared with those from image analysis.

### Detection with LSPR Spectroscopy

**Figure 2:**
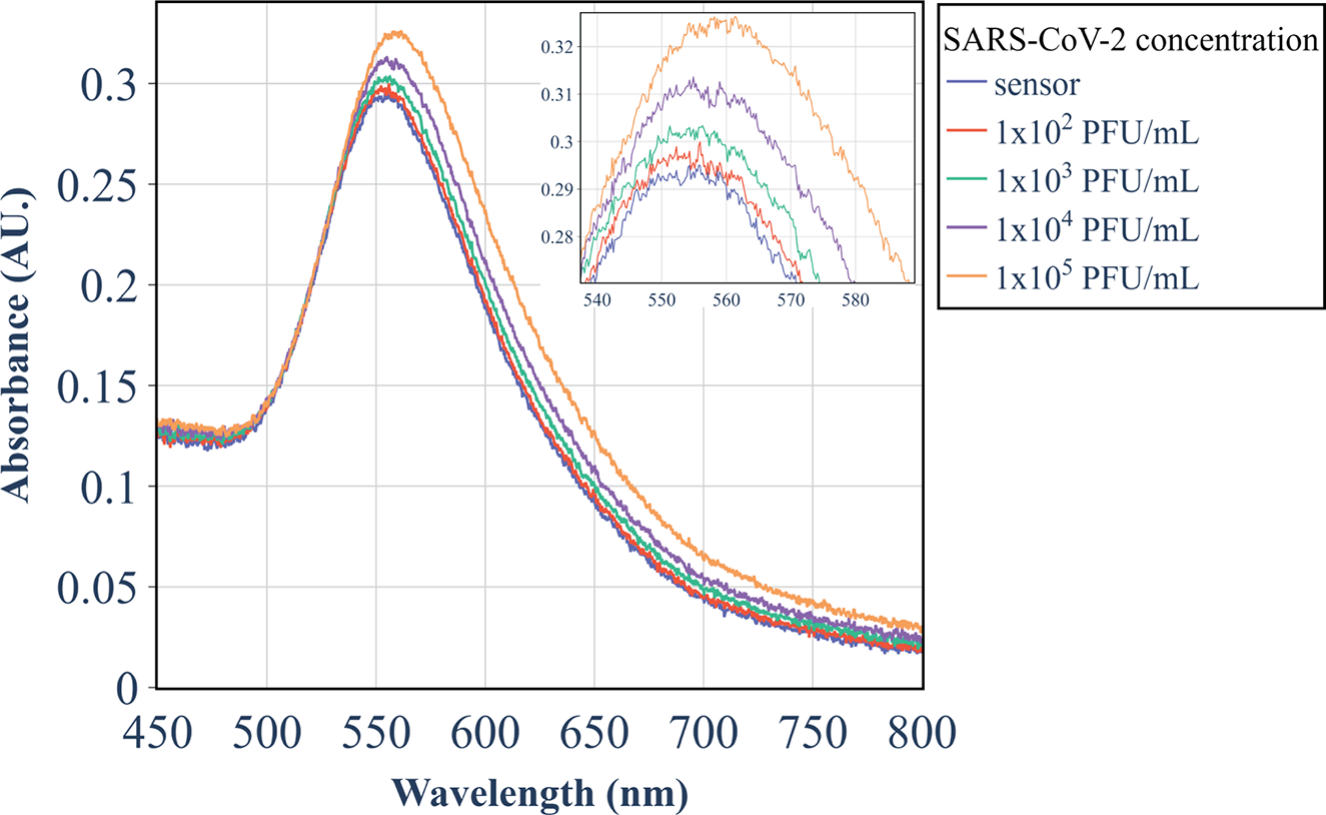
LSPR spectra for the immunosensor before and after positive tests with different concentrations of the SARS-CoV-2 virus.

Detection was performed with samples of various concentrations of inactivated SARS-CoV-2 virus, inactivated RSV, and blank tests with the PBS/MgCl_2_ buffer. The UV–vis LSPR spectra of the immunosensors were acquired before (state = probe or sensor) and after the detection tests. The changes in the spectra were examined with 10 features extracted from the LSPR band, as follows: wavelength and absorbance of the peak, fwhm, inf1, inf2, and valley (see Figure S4 in the Supporting Information). Figure 2 shows the spectral changes after tests with various concentrations of the SARS-CoV-2 virus. A redshift of the LSPR band was observed with increasing concentrations owing to the increase in the refractive index of AuNIs resulting from the adsorption of SARS-CoV-2 virus particles. Such adsorption occurs through the binding of spike-S1 proteins on the virus outer membrane and the anti-SARS-CoV-2 mAb monoclonal antibodies immobilized on the immunosensor.

**Figure 3:**
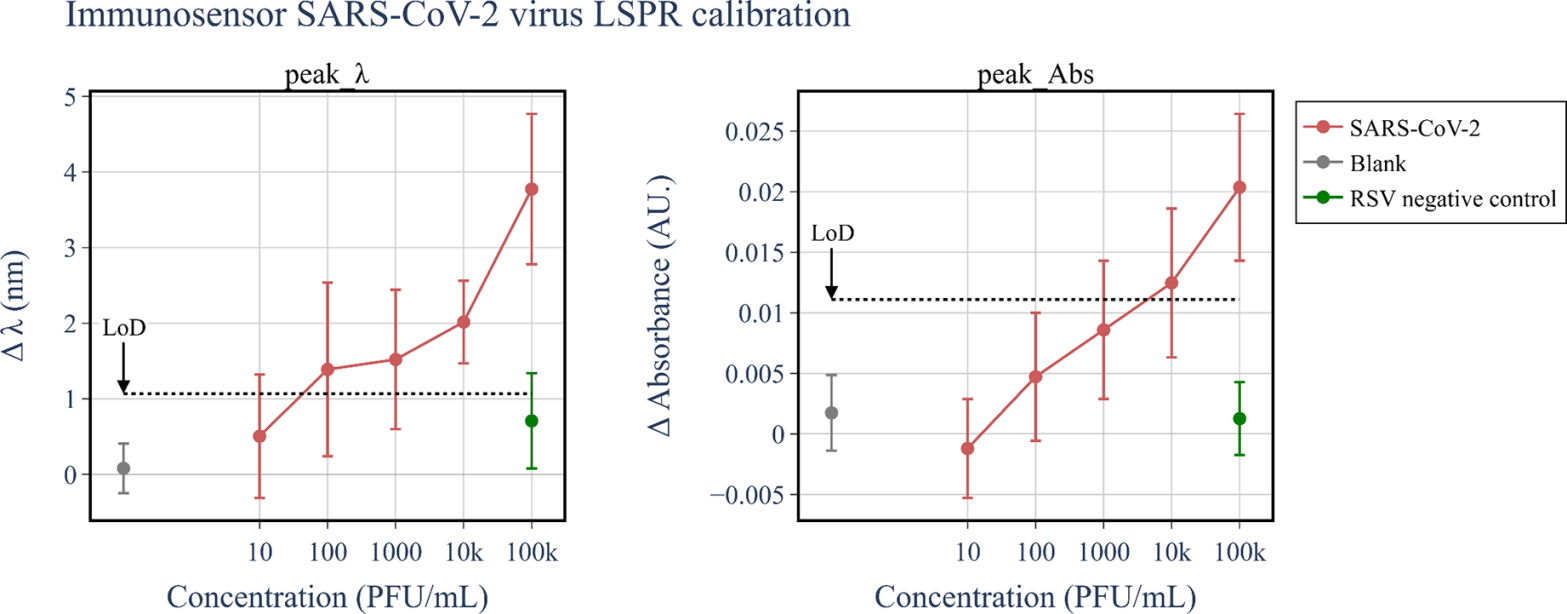
Calibration of the LSPR response in the positive tests for the features peak_λ and peak_abs (red dots). We included in the respective graphs the responses in the blank control tests (gray dots) and the negative control tests with the RSV virus (green dots).

The most distinctive response of the immunosensor was observed for the feature Peak_λ, which is illustrated in Figure 3 together with Peak_abs. The red dots represent the mean and standard deviation of LSPR responses in the positive tests with different concentrations of the SARS-CoV-2 virus. The green dot corresponds to the negative control tests with RSV virus particles, while the blank tests obtained with PBS/MgCl_2_ buffer are represented by the gray dot. We estimated the detection limit for features Peak_λ and Peak_abs according to IUPAC standards as being the mean plus 3× the standard deviation of the responses in the blank tests performed on 20 different sensors. The signal level of the LoD was 1.1 nm and 0.011 AU (absorbance units) for Peak_ λ and Peak_abs, respectively, shown on dashed lines in Figure 3. The mean response of Peak_λ for 1 × 10^3^ PFU/mL SARS-CoV-2 is above the LoD signal, but with a standard deviation below the detection level. Thus, we are unable to detect SARS-CoV-2 at concentrations below or equal to 1 × 10^3^ PFU/mL. For Peak_abs, the response was less sensitive, yielding a higher LoD. The deviations in the measurements may depend not only on the errors on the detection process but also on the differences in sensitivity between different plasmonic immunosensors of the same batch. The plasmonic substrates used in this study have a characteristic dispersion of 61 ± 21 nm in AuNI diameter and 52 ± 27 nm for interparticle distances (edge–edge) in the same batch (Figure S1 in Supporting Information). Figure 3 also serves to demonstrate the specificity of the immunosensor. The LSPR responses for features Peak_λ and Peak_abs in the tests with the RSV virus are below the detection level and close to the signal levels in the blank control tests. The specificity is higher when Peak_abs is considered since both the mean and standard deviation were below the corresponding LoD. It is worth noting that the LoDs estimated for the other LSPR features were higher, as indicated in Figure S3 of the Supporting Information.

**Figure 4:**
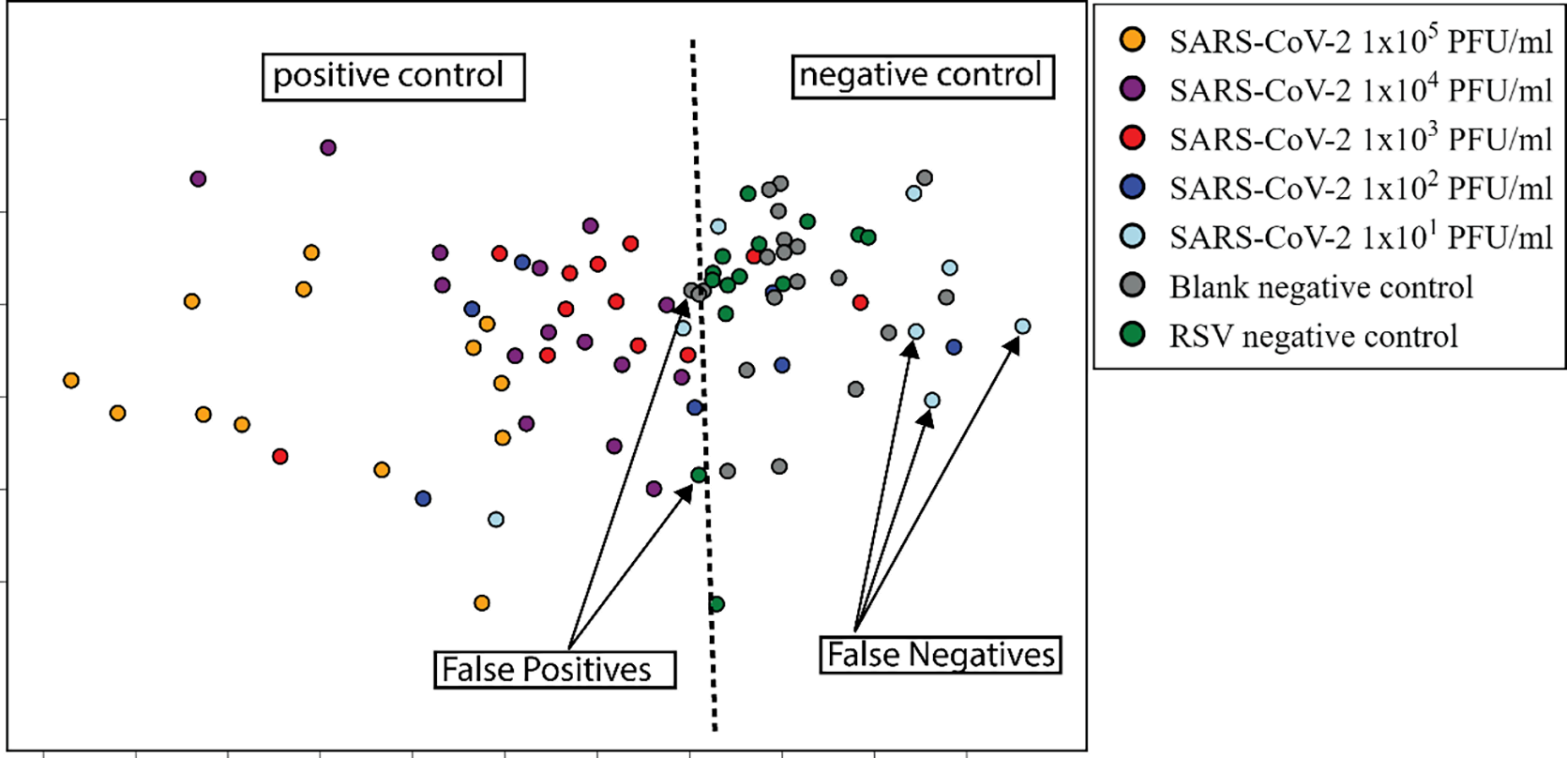
2D IDMAP projection of the 10 features extracted from the LSPR spectra in the tests performed. Each dot represents a measurement, and each type of test was marked with a different color. For heuristic analysis purposes, we included a dotted dividing line separating the regions that contain mostly positive tests from those that contain mostly negative control tests.

We also analyzed the immunosensor specificity taking into account the 10 features extracted from the LSPR spectra using the Interactive Document Mapping (IDMAP) technique^26,27^ to reduce the dimensionality. Figure 4 shows the 2D projection of the data where each dot represents an LSPR spectrum. Different colors were used to identify different SARS-CoV-2 concentrations and control tests. A dashed line was included to help distinguish the data points. Positive controls with higher concentrations (1 × 10^5^ and 1 × 10^4^, and most of the tests at 1 × 10^3^ PFU/mL) were projected on the left side. The points corresponding to positive control with lower concentrations (1 × 10^1^ and 1 × 10^2^, and some tests with 1 × 10^3^ PFU/mL concentration) were projected on the right side in the same region of the negative control tests. Therefore, these low-concentration samples could be considered as false-negative results; one point for the RSV tests was projected on the left side of the line, being considered as a false-positive result. The arbitrarily positioned dividing line allows us to analyze the specificity of the plasmonic immunosensor and its limitations when LSPR spectroscopy is used for detection. We did not apply machine learning methods to classify the samples because the amount of LSPR data is limited.

### Classification of Immunosensor Images Using Computer Vision and Machine Learning

We acquired 858 optical microscopy images (400× magnification) of the plasmonic immunosensors after the tests with SARS-CoV-2 virus at concentrations from 1 × 10^–4^ to 1 × 10^5^ PFU/mL, blank tests with pure PBS/MgCl_2_, control tests with RSV virus at concentrations from 1 × 10^3^ to 1 × 10^5^ PFU/mL, and the images of the class Probe corresponding to the images of the immunosensor before the tests. The number of images and the classes are detailed in Table 1. Some microscopy images of the immunosensors are included in Figure S6 of the Supporting Information. We employed 17 computer vision methods to extract features from each image, 5 of which are handcrafted: LBP, CLBP, GLCM, GLDM, RGB5D-LBP, and 12 are based on CNN: DenseNet121, EfficientNetV2_B0, EfficientNetV2_B1, EfficientNetV2_M, EfficientNetV2_S, MobileNet, MobileNetV2, MobileNetV3 small, ResNet18, ResNet34, VGG16, and VGG19.

**Table 1:** Distribution of Immunosensor Images for the Tests, Concentrations, and Classes

test, conc (PFU/mL)	class	*N*	ML distinguishable	2-Class	N_binary
SARS-CoV-2, 1 × 10^5^	CoV(5)	43	distinguishable	positive	211
SARS-CoV-2, 1 × 10^4^	CoV(4)	40	distinguishable	positive	
SARS-CoV-2, 1 × 10^3^	CoV(3)	21	distinguishable	positive	
SARS-CoV-2, 1 × 10^2^	CoV(2)	20	distinguishable	positive	
SARS-CoV-2, 1 × 10^1^	CoV(1)	21	distinguishable	positive	
SARS-CoV-2, 1 × 10^0^	CoV(0)	66	distinguishable	positive	
SARS-CoV-2, 1 × 10^–1^	CoV(−1)	40	not distinguishable		
SARS-CoV-2, 1 × 10^–2^	CoV(−2)	60	not distinguishable		
SARS-CoV-2, 1 × 10^–3^	CoV(−3)	42	not distinguishable		
SARS-CoV-2, 1 × 10^–4^	CoV(−4)	40	not distinguishable		
sensor, probe	probe	286	distinguishable from the positive classes	negative	501
blank, PBS/MgCl_2_	blank	140	distinguishable from the positive classes	negative	
RSV, 1 × 10^5^	RSV	39	distinguishable from the positive classes	negative	
RSV, 1 × 10^4^	RSV	16	distinguishable from the positive classes	negative	
RSV, 1 × 10^3^	RSV	20	distinguishable from the positive classes	negative	

The ML algorithms LDA, KNN, SVM, and RF were trained to classify the images in multiclassification and binary classification problems. The combination of all feature extraction methods with each ML classifier provides a set of 48 models based on CNN feature extraction and 44 models based on handcrafted feature extractors. Hence, a total of 92 image classification models were compared. We evaluated all models with cross-validation (stratified fivefold, repeated three times), and the mean and standard deviation of the metrics were calculated. The pipeline and details of this method are listed in Figure 1. The metrics used to analyze the performance of the models in multiclassification are accuracy and F1 score. To determine which model performs better in detecting the SARS-CoV-2 virus, we consider the accuracy score. The accuracy metric describes the model’s ability to differentiate the samples among all the classes considered in the training and prediction. The F1 score metric is defined as the harmonic mean between the precision and recall. This metric is more representative in the evaluation of the multiclassification problems since it considers both the recall and precision. It accounts for the performance of the model in distinguishing each individual class. The F1 score of the negative class can be used as a proxy of the selectivity or the ability of the sensor to predict the negative measurements correctly (minimized false positives) and not attribute the positive tests to the negative class (minimizing false negatives).

The image classification models were initially trained and evaluated for the multiclass problem considering all 10 positive tests, with the following classes: CoV(−4), CoV(−3), CoV(−2), CoV(−1), CoV(0), CoV(1), CoV(2), CoV(3), CoV(4), and CoV(5), corresponding to the SARS-CoV-2 dilutions from 1 × 10^–4^ to 1 × 10^5^ PFU/mL, and a negative class composed by merging the tests Blank, RSV (the three dilutions), and Probe in the same negative class. This 11-class problem was a hard training task for all of the models. The difficulty to classify the positive class concentrations is evidenced by the low values of accuracy and F1 scores in Table 2. The maximum accuracy among the 92 models tested was only 77.1 ± 2.4% for the feature extractor based on the CNN model MobileNetV3_small with the SVM classifier (model MobileNetV3_small + SVM). This may be explained by the inclusion in the data sets of very low concentrations, which may be below the limit of detection using image analysis. We recall that the LoD was 1 × 10^3^ PFU/mL when the LSPR spectra were used, corresponding to class CoV(3).

**Table 2:**
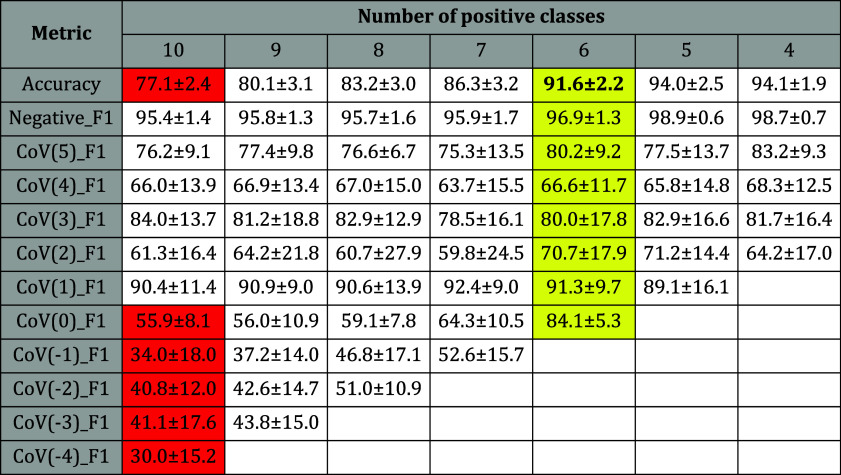
Metrics for the Multiclassification Using MobileNetV3_small + SVM Considering Different Numbers of Positive Classes (10, 9, ···, 4) and 1 Negative Class

In an attempt to determine the lowest concentration that could be correctly classified by the combination of image analysis and ML, we performed multiclassification tasks in which we progressively eliminated the lowest concentrations. The results of the multiclassification tasks are given in Table 2. The original multiclassification problem contains 11 classes: 10 positive classes (SARS-CoV-2 dilutions from 1 × 10^–4^ to 1 × 10^5^ PFU/mL) and 1 negative class. The accuracy and the F1 scores for each class for the original multiclass problem are included in column 10 (number of positive classes). Removing the lowest concentrated class, CoV(−4), corresponding to the dilution 1 × 10^–4^ PFU/mL, we trained and compared the 92 models to classify 9 positive classes (dilutions from 1 × 10^–3^ to 1 × 10^–5^ PFU/mL) and 1 negative class. The best accuracy and F1 scores for each class are included in column 9 (number of positive classes) and so on until removing class CoV(1), corresponding to the SARS-CoV-2 with concentration 1 × 10^1^ PFU/mL. In this case, we trained the models to classify the four positive concentrations, from 1 × 10^2^ to 1 × 10^5^ PFU/mL, and the negative class. In all multiclassification tasks, the highest-performing model was the MobileNetV3_small + SVM. The accuracy of this model increased from 77.1% for 10 positive classes to 91.6 and 94.0% for 6 and 5 positive classes, respectively. The F1 score of the negative class (Negative_F1) measures the performance of the model to distinguish the negative control tests. It did not vary with the number of positive classes considered; it was ca. 95%, which confirms the selectivity of the plasmonic immunosensor using optical microscopy and image classification with ML. In contrast, the F1 score of the positive classes was consistently low for the concentration 1 PFU/mL (class CoV(0)) and below. Together with the low accuracies for these low concentrations, one may conclude that they cannot be distinguished by using the image classification model. From the analysis of the accuracy and F1 scores for the various concentrations in Figure S10 in the Supporting Information, we infer that the minimum concentration distinguishable is either 1 or 1 × 10^1^ PFU/mL, class CoV(0) or CoV(1), respectively.

The performance in classification can be visualized in the confusion matrices in Figure 6, obtained for the best model (MobileNetV3_small + SVM) trained with different numbers of positive classes. These confusion matrices were constructed by selecting training and validation sets for which the accuracy is close to the average accuracies (Table 2). The blue-colored cells out of the principal diagonal represent the false positives and false negatives in the predictions. Misclassification occurs in positive classes corresponding to virus concentrations below 1 × 10° PFU/mL, class CoV(0).

**Figure 5:**
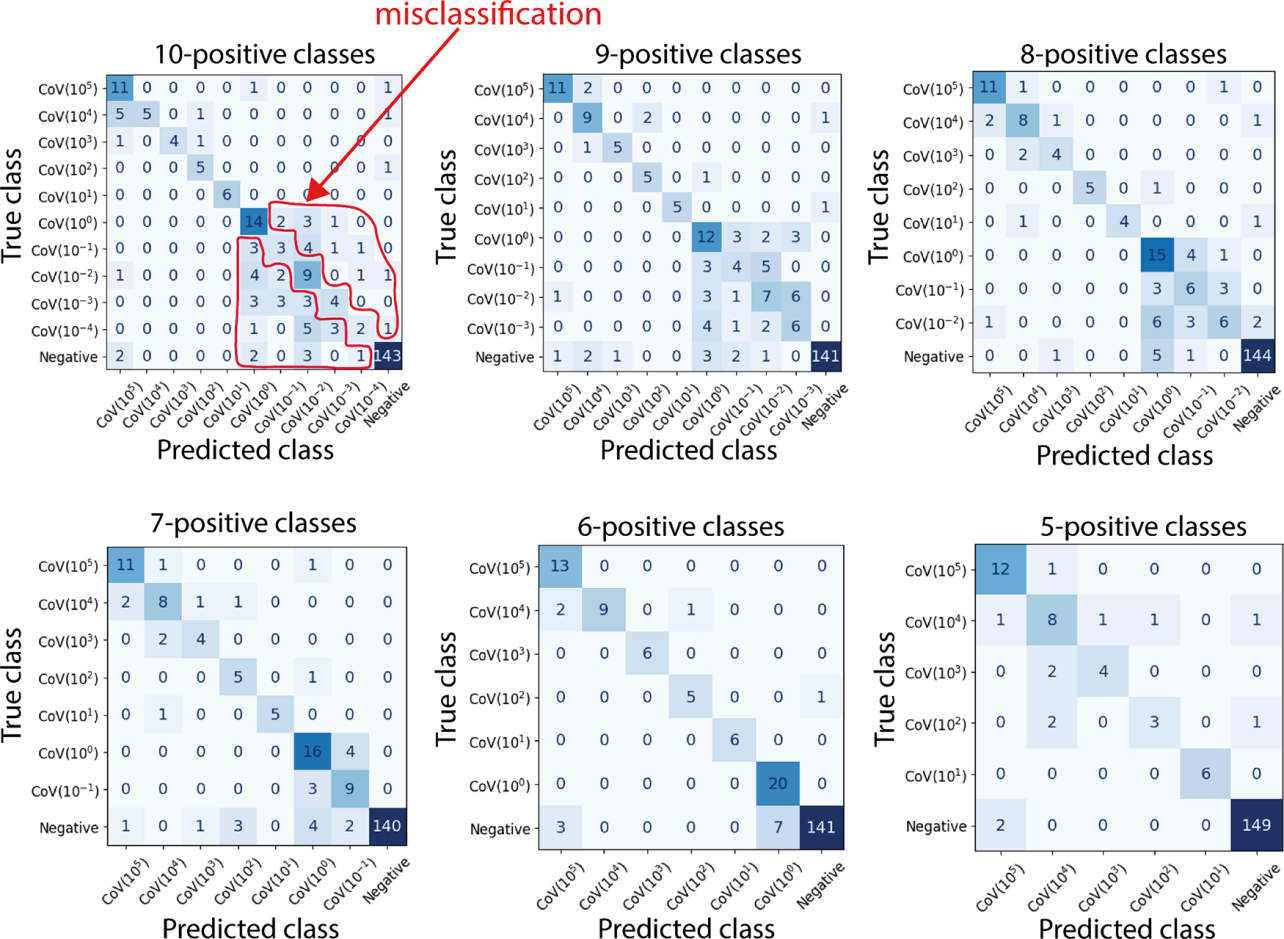
Confusion matrices obtained with the best model MobileNetV3_small + SVM, trained with different numbers of SARS-CoV-2 positive test dilutions, by removing the most diluted. The misclassification in the region of lower dilutions is seen in the blue-colored cells out of the principal diagonal of the matrices when the most diluted concentrations were considered. By removing the tests with smaller concentrations, the training process is easier, and the confusion matrix reveals a high performance for image classification of the different concentrations and in the negative tests. The confusion matrix of the problems containing six positive classes and one negative class corresponds to the classification performance of the best model trained with images of the sensor in the tests with the SARS-CoV-2 dilution from 1 × 10^0^ to 1 × 10^5^ PFU/mL. The six-positive class confusion matrix displays considerable improvement in the classification, and these performances were used to determine the minimum concentration distinguishable with image analysis for the plasmonic immunosensor.

**Figure 6:**
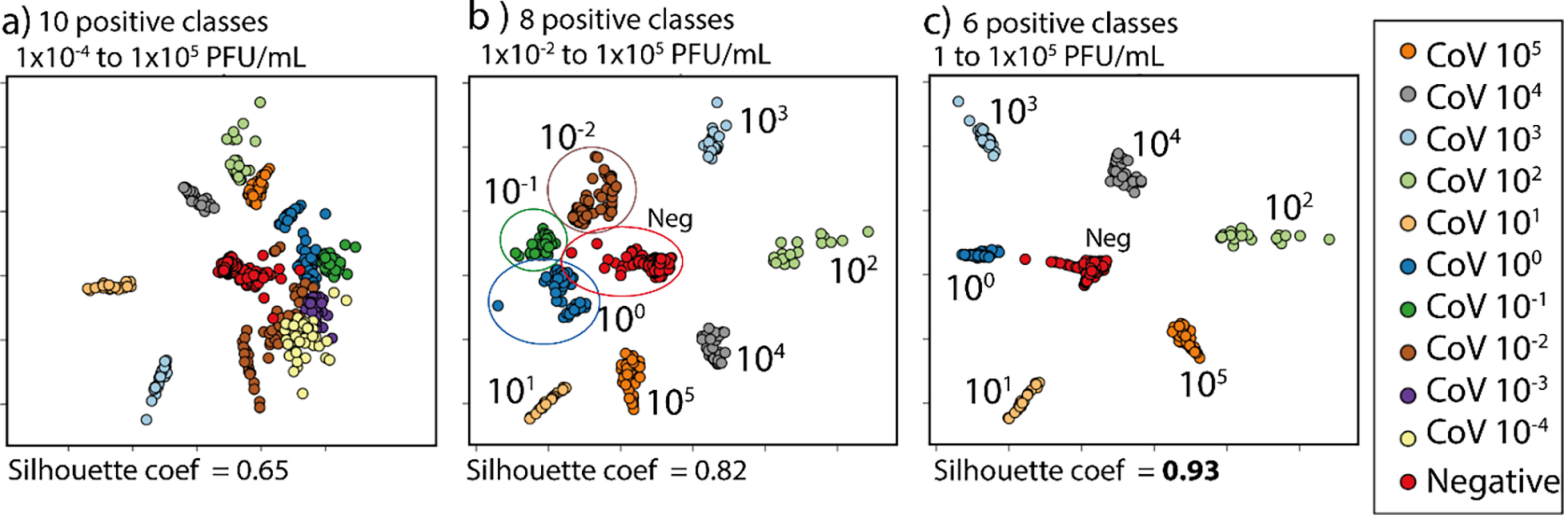
IDMAP of the predicted probabilities obtained from the MobileNetV3_small + SVM. (a) Training the model with 10 positive classes and 1 negative class, corresponding to the concentrations from 1 × 10^–4^ to 1 × 10^5^ PFU/mL. (b) Considering only eight positive classes and the negative class in the concentrations between 1 × 10^–2^ and 1 × 10^5^ PFU/mL. (c) IDMAP projection of the predicted probabilities with the model trained with the concentrations from 1 × 10° to 1 × 10^5^ PFU/mL (six positive classes) and the negative class. In the IDMAP (a, b), the low silhouette coefficient and the intersection between the clusters corresponding to lower concentrations indicate that concentrations were considered that cannot be distinguished. For concentrations at and above 1 PFU/mL, there are a clear separation and a high silhouette coefficient value of 0.93.

The determination of the minimum concentration distinguishable with the ML and image analysis method can be confirmed using IDMAP to visualize the predicted probabilities for all samples of the data set. The best model was trained with all samples, and then the trained model was applied to perform predictions for all samples in the data set, including those used for training and testing. Here, we did not make a train/validation split because we want to visualize how the model learns with the instances rather than make a prediction. The predicted probabilities for a given sample are an *n*-dimensional vector where *n* is the number of classes considered in the training. For example, considering six positive classes and one negative class in the training, the predicted probabilities are represented by a seven-dimensional vector with the probabilities of the sample belonging to each class. The sample’s predicted class is the class with the highest predicted probability value. We applied the predicted probabilities for all samples with the IDMAP method and created a 2D map that represented the samples as points in clusters associated with the predicted classes. The true classes of the samples are represented by distinct colors. Figure 6a–c shows IDMAP projections of the predicted probabilities obtained from the model MobileNetV3_small + SVM trained with 1 negative class and 10, 8, and 6 positive classes, respectively. When all concentrations from 1 × 10^–4^ to 1 × 10^5^ PFU/mL were used in the training, the model cannot distinguish the smallest concentrations in Figure 6a, leading to a silhouette coefficient of 0.65, as expected from the results in Figure S10 and Figure 5. Figure 6b shows a higher distinguishing ability, with an increased silhouette coefficient of 0.82, for concentrations starting at 1 × 10^–2^ to 1 × 10^5^ PFU/mL (eight positive classes). The overlap in Figure 6b also occurs when the concentration 1 × 10^–2^ PFU/mL is removed (see Figure S7 in the Supporting Information). A very high silhouette of 0.93 was obtained for the IDMAP plot for concentrations between 1 × 10^0^ and 1 × 10^5^ PFU/mL, where the classes are projected in well-separated, concise clusters. The silhouette coefficients of the IDMAP projections for different numbers of positive classes are shown in Figure S8, along with the IDMAP projections in Figure S7 of the Supporting Information. In conclusion, the minimum concentration that can be distinguished through image classification and immunosensing is 1 PFU/mL.

It is worth mentioning that the handcrafted models led to lower performances than the CNN models. The highest accuracy with a handcrafted feature extractor was 84.1 ± 2.3% for RGB5D-LBP combined with the RF classifier, owing the 30th best performance ranking when compared to all the 92 models. Full details of the results with these handcrafted models are shown in Table S2 and Figure S5 in the Supporting Information. The results obtained for multiclassification and the classification of tests with concentrations as low as 1 PFU/mL should be interpreted with caution. It may not be interpreted as the LOD. There is no standard definition to determine the LOD for detection methods based on ML classification. The results just demonstrated the ability of the AI model to learn to distinguish the SARS-CoV-2 tests in the data set available in this study. We also trained and evaluated the 92 image classification models for binary classification. The six positive concentrations of SARS-CoV-2 virus, from 1 to 1 × 10^5^ PFU/mL, were included in a single positive class, and the control tests Blank, RSV, and Probe were included in the negative class. The number of images and the description of the classes are given in Table 1. In binary detection, we evaluated the models using the accuracy, recall (sensitivity), precision, true negative rate, and the negative predictive value. The best binary classification model was the CNN-based MobileNetV3_small + LDA that demonstrated an accuracy of 96.5 ± 1.6%, recall of 92.5 ± 4.1%, and negative predictive value of 96.9 ± 1.6%. Among the handcrafted methods, the best performance was obtained with the model RGB5D-LBP + RF with an accuracy of 90.8 ± 1.9% (29th ranking position among all models compared). The complete results of the binary classification models are included in the Supporting Information. The accuracy for binary classification was higher than that for multiclassification, as expected.

**Figure 7:**
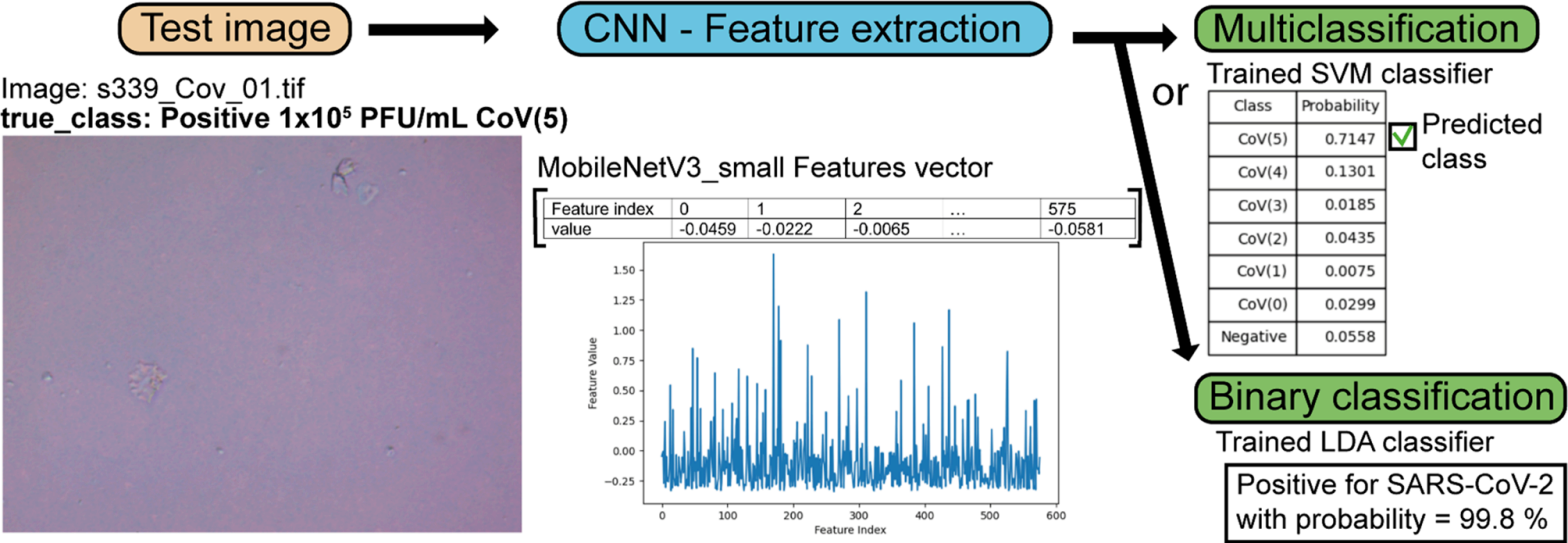
Pipeline of a test with a given image of the SARS-CoV-2 detection and concentration estimation using image classification trained models. The model MobileNetV3_small + SVM was applied to predict the class of a test image, and the model MobileNetV3_small + LDA was used to predict between positive and negative. We illustrate the image features in a bar plot. This vector was applied to the trained ML algorithms SVM and/or LDA. The probability for the predicted class to be CoV(5) is shown in the tables. It is 71.5% for the SARS-CoV-2 test with the concentration 1 × 10^5^ PFU/mL in the multiclassification task. The match of the predicted class with the true class demonstrated the accuracy of the image classification model. The binary model predicts correctly the class of the test image with 99.8% predicted probability.

A detection test in a chosen image using image classification with ML is illustrated in Figure 7 for the true class of immunosensor image corresponding to SARS-CoV-2 with concentration 1 × 10^5^ PFU/mL. The features extracted using the MobileNetV3_small, returning a vector with 576 features, are represented in the bar plot. We can perform multiclass classification using the trained SVM algorithm yielding the predicted probabilities for each class. We can also perform the binary classification using the trained LDA algorithm. In this example, the highest probability is 71.5% for the class CoV(5), determining correctly the predicted class of the test image, while using the binary model, the result was positive with 99.8% predicted probability.

In subsidiary analysis, we noted that using the RGB images led to slightly higher accuracy for the CNN and handcrafted methods, as described in the Supporting Information with the discussion of the results shown in Table S5. The small difference in performance indicates that texture and not color is the main contributor for distinguishing the images of immunosensors exposed to the different concentrations of SARS-CoV-2.

The antigen detection kits based on flow assays, used for the rapid qualitative detection of the SARS-CoV-2 virus in nasopharyngeal fluid, have a limit of detection (LOD) in the range from 10^2^ to 10^3^ PFU/mL.^47,48^ In comparison, RT-PCR assays demonstrate LOD values between 10^–2^ and 1 PFU/mL.^49^ RT-PCR assays are capable of detecting very low levels of viral plasmid genes, although the ratio of RNA copies to PFU can vary.^50^ Our sensor demonstrated the ability to detect SARS-CoV-2 at concentrations as low as 1 PFU/mL, achieving performance comparable with the most sensitive sensors reported in the literature.^13−17^ However, the tests were conducted only with inactivated samples diluted in a PBS/MgCl_2_ buffer. In clinical applications with real samples, the test solutions are more complex. Therefore, our sensor could be further trained using clinical samples.

In PoC applications, it is relevant to consider the computational cost of the models, as the processing time and computational resources required can increase the cost of testing. In a real application, the ML model is already optimized, and the test can be performed with the analysis of a few images of the immunosensor. The processing times were estimated for all models considering the image processing, feature extraction, and classification. In the classification part, with a trained ML model, the load and classification of three images were performed in less than 0.2 s (results not shown) for the ML models compared (LDA, KNN, SVM, and RF). The image preprocessing and feature extraction task are the main contributors for the detection processing time (see Tables S6 and S7). The processing times and the number of features for the handcrafted texture extractors, running using only the CPU, were compared in Table S6. For the CNN-based models, the number of features extracted, the number of FLOPs (“Floating Point Operations”), and processing time were compared in Table S7. The CNN models were compared by running with a GPU, NVIDIA Tesla T4 GPU with 16 GB VRAM, provided by Google Colab 40. The execution time of the MobleNetV3_small feature extractor was 0.18 s per image. The handcrafted method RGB5D-LBP, using only the CPU, runs in 9.3 s per image. Although they have exhibited lower performance, handcrafted methods require less computational power, potentially rendering them more suitable for use in embedded systems or portable computers. On the other hand, methods based on convolutional neural networks have achieved superior performance but demand greater computational power.

In this study, we trained models to classify sensor images in their original format as captured by the microscope, with dimensions of 1920 × 2640 pixels, without any preprocessing. CNN algorithms were used as feature extractors with the first input layer matching the original image size. Typically, when using CNN pretrained models, the images are scaled to 224 × 224 pixels, as CNN algorithms are pretrained on the ImageNet^40^ database with this size. However, we opted to use the original image format because in this configuration, the pixel distance is approximately 86 nm, which is comparable to the AuNI average diameter (approximately 61 nm), as seen in Figure S1. The extracted texture features capture the patterns of intensity fluctuations between pixels, which may result from the affinity and other interactions between the analyte and the probe molecules on the sensor surface. The possibility of using lower magnifications and even cellphone images is currently under investigation by our group. Another avenue of exploration is the use of deeper CNN architectures, such as ResNet50, ResNet101, and EfficientNetV2_L, among others. Additionally, customizing the number of layers in a CNN architecture could help determine the optimal model depth for extracting the most relevant features. We employed traditional ML models commonly used for small data sets and in texture analysis, providing satisfactory results. If a larger data set was available, training could be also applied to the CNN architectures, which could potentially lead to even more robust models for virus detection. However, it is important to note that in biosensor research, the availability of samples is often limited. Despite these constraints, excellent results in image classification and, consequently, diagnosis can still be achieved with a limited data set, as demonstrated in this study. Combining feature extractors with classifiers to create ensemble models may also improve classification performance. Several image analysis strategies can be further investigated in this context.

## Conclusions

We have shown that diagnostics using immunosensors can be made by applying machine learning and computer vision algorithms in analyzing optical microscopy images taken from the sensors before and after exposure to biological samples. This was demonstrated with a plasmonic immunosensor made with AuNI/glass substrates in detecting inactivated SARS-CoV-2 virus particles. It is significant that a very low concentration of 1 PFU/mL could be distinguished from higher concentrations and negative controls with the ML-based image analysis, while the limit of detection (LoD) was 1 × 10^3^ PFU/mL for sensing using LSPR spectroscopy. To the best of our knowledge, no other studies in the literature employ the same methodology as ours in the detection of plasmonic biosensors.

The high performance mentioned above was obtained by testing 92 image classification models with feature extraction made either with deep learning and CNN (48 models) or with handcrafted methods (44 models). The CNN models yielded a higher performance than the handcrafted ones. As for the ML algorithms, the highest performance was obtained with the model MobileNetV3_small + SVM for multiclassification and mobileNetV3_small + RF for binary classification. We also compared image analysis approaches including color features and without them. We noted that the main contribution to a correct classification comes from texture, which has important implications since there are many efficient methods to classify images based on texture. One can envisage the extension of the approach employed here to any type of immunosensor or genosensors. There is ample evidence in the literature that detection with these types of sensors is governed by adsorption processes that will affect texture.^3,51^ Here, we demonstrated that changes in images at the micrometer scale can be detected, but it remains to be checked whether the method can be extended to images taken with smartphone cameras. Moreover, the validity of the present analysis, which incorporates CNN architectures and machine learning classification, is not limited to the detection of SARS-CoV-2 but can also be applied to other analytes.

The main challenge for achieving a diagnostic system based on optical microscopy image analysis with ML, e.g., for PoC applications, is the amount of data required for training the models. This requires low-cost sensors, which can be obtained with the AuNI/glass plasmonic substrates used here.

## Data Availability

This material is available free of charge via the Internet at http://pubs.acs.org. The data set of optical microscopy images of the sensors and the tables with the features extracted with the computer vision models were included in the repository https://github.com/praoiticica/COVID-plasmonic-sensor-ML and in the repository Mendeley Data, V1, doi: 10.17632/z4js67w5vc.1.

## Supporting Information Available

FESEM image of plasmonic substrate; additional experimental details and supplementary results of LSPR spectrum measurements; description of the IDMAP method and supplementary results; theoretical description of the machine vision models used in the work; theoretical description of the machine learning algorithms used for classification; definition of ML metrics used; optical microscopy images of the plasmonic sensors; supplementary results of the sensor image classification; tables with the processing times for each feature extractor model (PDF)
