# Supplementary material for: Using Machine Learning and Optical Microscopy Image Analysis of Immunosensors Made on Plasmonic Substrates: Application to Detect the SARS-CoV-2 Virus
Source: ACS Sens. 2025 Feb 17;10(2):1407–18. doi: 10.1021/acssensors.4c03451 (PMC11877505; doi:10.1021/acssensors.4c03451)
Supplement: Supplementary file 1 — se4c03451_si_001.pdf [file se4c03451_si_001.pdf]

Supplementary Material

Using machine learning and optical microscopy image analysis of immunosensors made on plasmonic substrates: Application to detect SARS-CoV-2 virus

Pedro R. A. Oiticica[1,2], Monara K. S. C. Angelim[3], Juliana C. Soares[1], Andrey C. Soares[2], José L. Proença-Módena[3,4], Odemir M. Bruno[1], Osvaldo N. Oliveira Jr[1*].

[1] São Carlos Institute of Physics (IFSC), University of São Paulo (USP), 13566-590 São Carlos, SP, Brazil.

[2] Nanotechnology National Laboratory for Agriculture (LNNA), Embrapa Instrumentação, 13560-970 São Carlos, SP, Brazil.

[3] Department of Genetics, Evolution, Microbiology and Immunology, Institute of Biology, University of Campinas, 13083-862, Campinas, SP, Brazil.

[4] Experimental Medicine Research Cluster (EMRC), University of Campinas, 13083-862, Campinas, SP, Brazil.

Corresponding author: chu@ifsc.usp.br

S.1. Plasmonic substrates morphology

Figure S1 shows the morphology of the AuNI/glass plasmonic substrates obtained with FESEM (Field Enhanced Scanning Electron Microscopy*)* using a Zeiss Sigma microscope (Carl Zeiss, Germany) equipped with the Gemini column. The particle analysis was made with python (v. 3.9) programs using the libraries SciPy (v. 1.9.3), scikit-image (v. 0.20.0) and scikit-learn (v. 1.2.2). This analysis included threshold segmentation followed by region properties estimations of the equivalent circular diameter, 1st nearest neighbor interparticle distances (edge-edge), particle density and the surface fraction covered by AuNIs. The average values and standard deviations were obtained considering different substrates in the same batch.

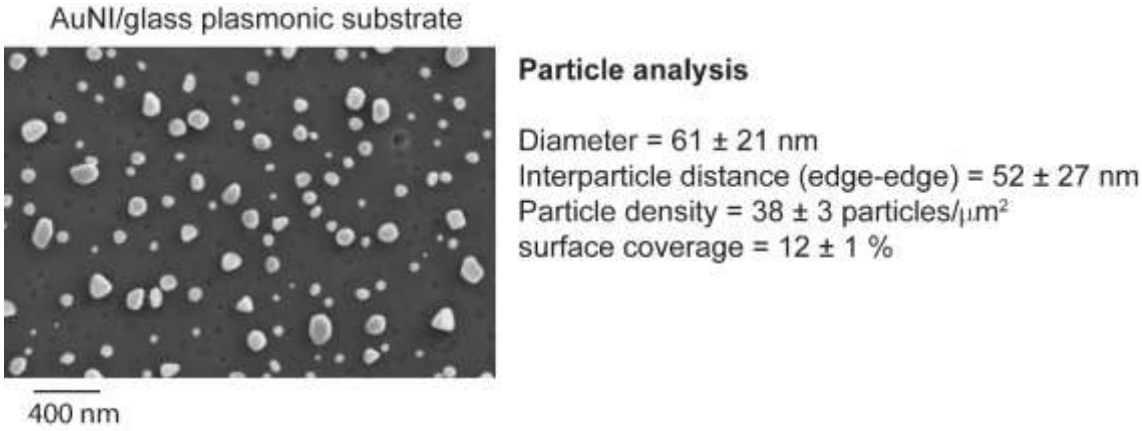

Figure S1. Morphology and particle analysis of the AuNI/glass substrates used for the immunosensor.

The plasmonic substrates have a characteristic texture due to the random distribution of particle sizes. All chips used in this work were previously selected in a single fabrication batch and in the LSPR spectrum. The selected chips have almost identical LSPR spectrum and therefore the distributions of AuNIs are similar. We fixed all possible parameters for the plasmonic substrates and image microscopy measurements to ensure that the machine learning classification is based on the differences in virus concentration and not from differences in sensor batches or imaging parameters.

S.2. UV-Vis LSPR measurements

Figure S2 shows the setup for measuring the LSPR UV-Vis spectra with the plasmonic immunosensor.

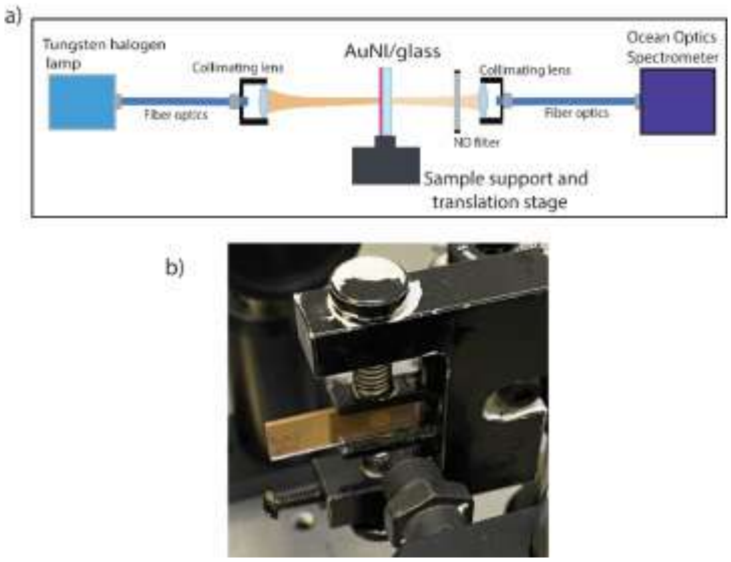

Figure S2. Setup for LSPR spectroscopy measurements a) Schematics of the fiber optic spectrometer. b) sample holder and probe light beam.

The LSPR responses for the LSPR features inf1, inf2, FWHM and valley are presented in Figure S3. The red dots correspond to the responses in the positive tests with different concentrations of SARS-CoV-2 virus. The blank tests were represented in the gray dot, while the negative control tests with RSV virus are represented by the green dots. The IUPAC detection limit LoD for different

features were represented by the black line separating the positive concentrations below the detection level.

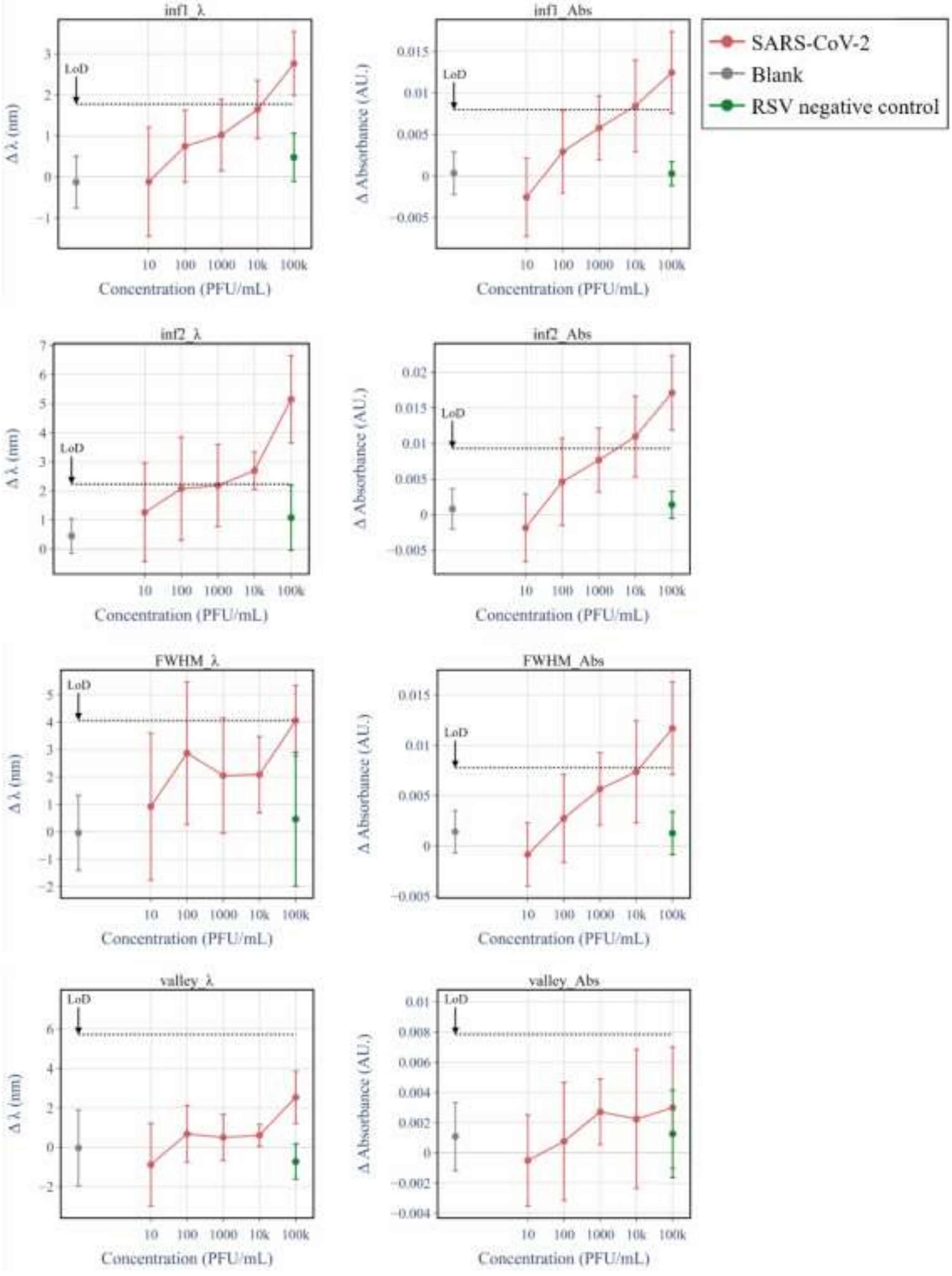

Figure S3. Calibration of the LSPR response in the positive tests for the features inf1, inf2, FWHM, and valley. The red dots represent the positive tests with different concentrations of the virus SARS-CoV-2. We included the responses from the blank control tests (gray dots) and the negative control tests with the RSV virus (green dots).The LoD level for the respective feature is presented in the black dotted line.

S.3. Pipeline of the feature extraction from LSPR spectra measurements

Programs in python were written to extract 10 features of the LSPR band characteristic of the AuNI/glass substrates. The codes use Numpy and Scikit-Learn libraries and the Peakutils package. The features are referred to as: Peak_λ, Peak_abs, FWHM_λ, FWHM_abs, inf1_λ, inf1_abs, inf2_λ, inf2_abs, valley_λ, valley_abs. Peak_λ and Peak_abs represent the wavelength and absorbance at the point of maximum absorbance. Features inf1_λ and inf1_abs correspond to the wavelength and absorbance at the inflection point located on the left side of the LSPR band. Inf2_λ and inf2_abs refer to the inflection point on the right side of the LSPR band. The full width at the half maximum (FWHM_λ) of the LSPR band is estimated based on the method proposed by Muri et.al.[1], considering the valley of the spectrum as the baseline. The feature FWHM_abs is the absorbance at the FWHM point. The features valley_λ and valley_abs correspond the point of minimum absorbance on the left side of the LSPR spectrum (between 400 – 550 nm) characteristic of the plasmonic substrates used in this study. Figure S4 shows the location points for each feature in the AuNI/glass LSPR spectrum.

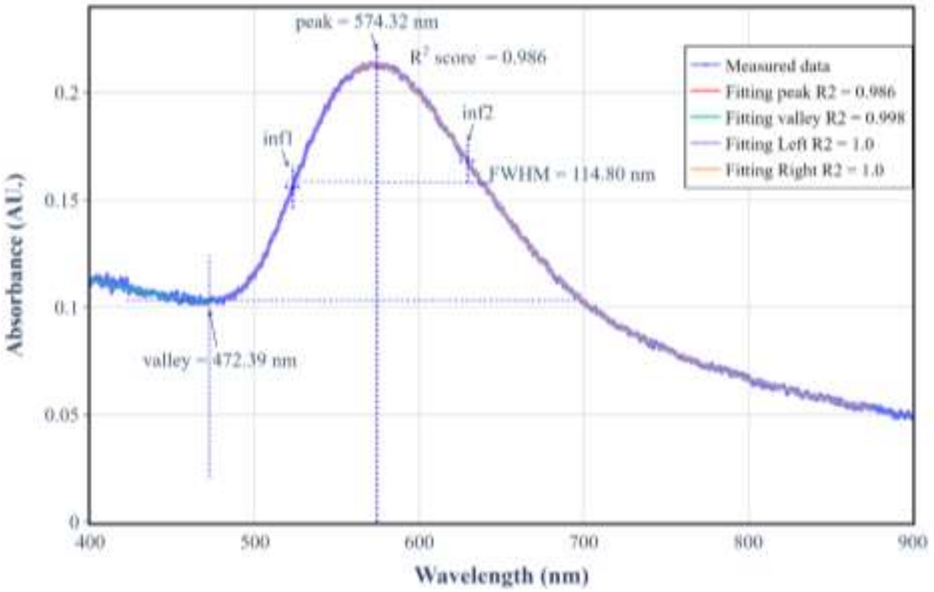

Figure S4. Extraction of LSPR features. The measured data and the fitting lines adjusted to estimate the features. The points corresponding to each feature were estimated for the AuNI/glass LSPR spectrum using programs in python.

S.4. Information visualization with IDMAP

The IDMAP ("Interactive Document Mapping") method[2,3] is used for reducing dimensionality and visualizing information by conserving the similarity between the experimental data in the original multidimensional space. This method projects a collection of documents or samples in an n-dimensional space to a smaller dimensional space using the FastMap dimensionality reduction technique[4], combined with the *force scheme technique*[5] to improve the position of the points in the iterative process. This technique is implemented in the PEx-Sensors software, available on the VICG portal (http://vicg.icmc.usp.br/vicg/tool/2/pex-sensors, accessed on April 10, 2024). It was applied to the analysis of biosensor data to project a set of spectra measurements to a 2D space for information visualization and cluster analysis using the silhouette coefficient.[6] IDMAP can be used to analyze multidimensional data, to evaluate selectivity, sensitivity, the presence of false positives, to identify patterns in the data that may indicate trends, faults, errors, spurious min/max values and clusters.[7,8] The IDMAP method performs dimensionality reduction by preserving the relative distances between pairs of samples in the original N-dimensional space, in the projected space. The distances between pairs of points in the projected space (which represent measurements or samples) are proportional to the similarity between them in the original space. Let us assume a set of N-dimensional data instances. The Euclidean distance $\delta(x_i, x_j)$ (other distance metrics can be employed) between any two samples $\boldsymbol{x}_i = (x_{i1}, x_{i2}, x_{i3}, ..., x_{in})$, and $\boldsymbol{x}_j = (x_{j1}, x_{j2}, x_{j3}, ..., x_{jn})$ in the original space is computed as a proxy for their dissimilarity. By minimizing the error of the equation S1 below we obtain a new representation of the samples on a 2D space given by $y_i = (y_{i1}, y_{i2})$ and $y_j = (y_{j1}, y_{j2})$, corresponding to Euclidean distances $\delta(y_i, y_j)$.

$$Error\_IDMAP = \frac{\delta(\boldsymbol{x}_i, \boldsymbol{x}_j) - \delta_{\min}}{\delta_{\max} - \delta_{\min}} - \delta(\boldsymbol{y}_i, \boldsymbol{y}_j) \qquad \text{S1}$$

where $\delta_{\max}$ and $\delta_{\min}$ represent the maximum and minimum distances between two instances of data in the data set, computed in the original space.

S.5. The handcrafted methods of computer vision

GLCM (*Gray Level Co-occurrence Matrix*)[9] is a traditional method of texture extraction proposed by Haralick in 1973. The co-occurrence matrix is obtained for all pairs of pixels, separated by a distance d and angle θ. We then estimate second-order statistical measures in the competition matrix. Each statistical measure is a feature extracted from the image.[10]

GLDM (*Gray Level Difference Method*): It is a method of texture analysis based on first-order statistical measures on the probabilities of gray level absolute difference values between pairs of pixels separated by a given distance δ. The image difference is calculated according to equation S2 by the absolute difference in gray level of pixels separated by $\delta = (\Delta x, \Delta y)$.

$$f_\delta(y,x) = \left| f(y,x) - f(y+\Delta y, x+\Delta x) \right| \quad \text{S2}$$

where $f(y,x)$ is the gray level of the image in row y and column x and the offsets Δy and Δx are integers and pixel distances. The number of levels of the absolute difference image will be the same as the number of gray levels of the original image (256 levels for uint8 type images). The normalized histogram of the GLDM image represents the probabilities or frequencies of occurrences for each possible absolute gray level difference value i. The features are obtained from the statistical measurements on the histogram of the image of absolute differences. Not all measures of the GLCM method can be applied to the GLDM method because it is given in a 1D probability matrix while GLCM has a 2D probability matrix. The measures used in GLDM are typically: contrast, ASM (Angular Second Moment), entropy, mean, and IDM (Inverse Difference Moment).[10] The feature vector is constructed by the concatenation of measurements obtained from various displacements with different scales and directions. For example, if we use the displacements (dx,dy): (1,0), (2,0), (3,0), ..., (8,0), (0,1), (0,2), (0,3), ..., (0,8), and estimate 5 measures on each GLDM

histogram corresponding to the displacements, we can build the feature vector by concatenating 16 offsets x 5 measures = 80 features to describe the image.

The LBP (Local Binary Pattern) method was developed by Ojala et al.[11] It consists of applying an operation to each pixel of the image, calculating the differences between the pixels located in a circular pattern of radius R and the central pixel, converting the result into a binary pattern. Thus, for each pixel a binary pattern is obtained and coded according to equation S3.

$$LBP_{riu2,P,R}(i,j)=\begin{cases}\sum_{0}^{P-1}\delta(g_p-g_c),\ if\ U(LBP)\le 2\\ P+1,\ if\ U(LBP)>2\end{cases} \qquad \text{S3}$$

$$\delta(gn-gc)=\begin{cases}1,\ g_n\ge g_c\\ 0,\ g_n<g_c\end{cases}$$

where g_c is the intensity of the central pixel, g_p is the intensity of the pixel at point p of the circular pattern of P points, R is the radius of the circular pattern, and U(LPB) is the number of discontinuities 01 or 10 of the resulting binary pattern. This rule for the number of discontinuities is an encoding format (U2). We can also consider the rotation invariant patterns (riu2) to encode the binary patterns or "textons" according to equation S3.

The method CLBP (*Complete Local Binary Pattern*) was developed by Zhenhua Guo et al.[12] in 2010 as an extension of the LBP[11] method. It consists of 3 components; the classical LBP, the difference magnitude component (CLPB_M) and a center gray level component (CLBP_C) given by equations S4 and S5.

$$CLBP\_M_{riu2,P,R}(i,j)=\begin{cases}\sum_{0}^{P-1}\delta\left(\left|g_p-g_c\right|-\ C1\right),\text{se}\ U(LBP)\le 2\\ P+1, se\ U(LBP)>2\end{cases} \qquad \text{S4}$$

$$CLBP\_C_{riu2,P,R}(i,j)=\begin{cases}\sum_{0}^{P-1}\delta\left(g_c-\ C2\right),\text{se}\ U(LBP)\le 2\\ P+1, se\ U(LBP)>2\end{cases} \qquad \text{S5}$$

where the magnitude component is calculated by the modulus of the difference between the pixel of the circular pattern and the central pattern minus the average of the values of the absolute difference for all pixels in the image, denoted by C1. The CLBP_C component is calculated in the same way by comparing the intensity of the central pixel with the C2 value given by averaging the intensity of the pixels throughout the image. The feature vector is obtained by the combination of the histograms of the components via simple concatenation or combined pixel by pixel. The number of features is given by the number of points P, the coding method used, and the method of combining the components. For example, the CLBP method with the encoding of type riu2, the number of neighbors P = 16 points, radius R = 2 pixels, and combination of components using the S_M/C method, results in (P+2) + (P+2) x 2 = 54 features. Using S/M/C encoding we would have (P+2) x (P+2) x 2 = 648 *features*. For other configurations, see the original reference of the method[12]. The LBP and CLBP methods are used to extract texture features from grayscale images.

The feature extractor RGB5D-LBP. We designed the feature extractor method RGB5D-LBP (RGB to 5 Dimensions Local Binary Pattern) used in this work. This method was written to extract features from RGB images. It was based on the MCLBP (Multiple Channels local Binary Pattern) algorithm proposed by Xin Shu et al.[13], and the LBP[11] method. The RGB5D-LBP combined the LBP of 3 channels (LBP_R, LBP_G, LBP_B) with two perpendicular components called LBP_Gx and LBP_Gy. The algorithm extracts the LBP features in a circular pattern using bilinear interpolation, according to the original LBP[11] method, and two perpendicular components LBP_Gx and LBP_Gy in a squared coordinate pattern. This pattern is pre-defined for each radius value and the number of points P is given by the radius R according to the equation P = 4+4R. The number of points in the LBP circular pattern is the same as the number of points considered in the perpendicular components, being P=8 for R=1, P = 12 for R=2 and P=16 for R=3. **Figure S5** shows the components of the RGB5D-LBP method using the parameters P=8 and R=1, for an image of the AuNI/glass immunosensor illustrating the three LBP components and the square coordinate pattern for the

perpendicular components LBP_Gx and LBP_Gy. The concatenation of the 5 components represents the features_vector (equation S6).

$$RGB5D-LBP\_vector=[LBP_R, LBP_G, LBP_B, LBP\_Gx, LBP\_Gy] \qquad S6$$

The number of features is given by the number of points P according to the equation S7

$$n\_features(P)=(P+2)+(P+2)+(P+2) \qquad S7$$

The RGB5D-LBP feature extractor differs from the MCLBP proposed by Xin Shu et al.[13] in the number of components, the pattern coordinates and the dimensionality of the feature vector. The method MCLBP contains 9 components (3 LBP components and 6 perpendicular components). The 3 channel LBP components use a square coordinate pattern where RGB5D-LBP applies circular pattern using bilinear interpolation. Moreover, in MCLBP the number of points considered in the squared LBP pattern is different from the number of points in the perpendicular components while in RGB5D-LBP the number of points in each component is the same.

The algorithm of the method RGB5D-LBP was written in cython language and implemented in a python module to optimize the computational efficiency and reduce the processing time. It uses the library scikit-image[14], the sub-modules Interpolation.pxd, Fused_numerics.pxd and the functions bilinear_interpolation and get_pixel3d.

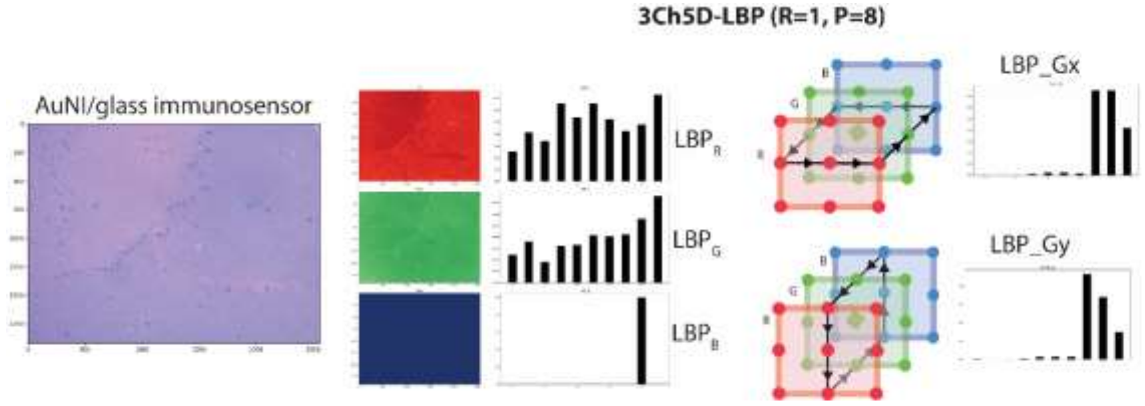

Figure S5. Application of the feature extractor RGB5D-LBP to an image of the AuNI/gass immunosensor and the 5 components of this method. It illustrates the square coordinate template used to extract the perpendicular LBP components LBP_Gx and BP_Gy, using the parameters P=8 and R = 1.

S.6. The CNN models used for image feature extraction

The CNN architectures employed here were used as feature extractors and the parameters were imported from models pre-trained on the ImageNet sub dataset[15] with 1.2 million images and 1000 classes.

The **VGG** architecture (Simonyan et al., 2014) was developed by the *Visual Geometry Group* and won the second place in the 2014 ILSVRC competition. Its main contribution was to demonstrate that architectures with a higher number of layers (up to 19 layers were tested) can improve performance for large-scale image classification. At the top of this model there is the classification layer composed of 3 dense layers with 4096, 4096 and 1000 nodes, respectively. The ReLu activation function is used in the hidden layers and the *soft-max* activation function is used in the classification layer to classify the 1000 classes of the ILSVRC – 2014. The filters applied to generate the convolutional layers have 3x3 pixels kernel. The architecture uses *max-pooling operation* (2x2, with stride 2) reducing the dimension of the layers by half in both dimensions and multiplying by two the number of filters folded in the next layer. In this work we used VGG with 16 and 19 layers (versions VGG-16 and VGG-19), removing the classification denselayer and applying GAP to result in a 512-dimension features vector.

The **ResNet** architecture (He et al., 2016) was the winner in the 2015 ILSVRC competition with the introduction of residual blocks as its main contribution. This

block allows for the stacking of more convolutional layers without degrading the performance of the model, causing the network to focus on learning the residuals instead of focusing only on the absolute value of the layer output. This approach contributes to solving the problem of *Vanishing Gradient* that is common in deep architectures with a very large number of intermediate layers. In this work, we used the ResNe-18 and ResNet-34 architectures, which contain 18 and 34 layers, respectively.

The **DenseNet** architecture (Huang et al., 2017) stands out for its dense connectivity, in which each layer is interconnected with all previous layers. This results in a direct and maximized flow of information across the network. Unlike ResNet models, *features* are not combined by summation before passing through a layer. Despite the dense interconnection approach, this network requires few parameters compared to other traditional CNN models, as it does not require relearning feature maps.[18]. In this work we used the DenseNet121 architecture, which is the lightest and generates the lowest number of features in the output (1024 *features*) compared to the others (DensNet169, DenseNet201, ...).

The **MobileNet, MobileNetV2 and MobileNetv3_small** architectures[19–21] were developed prioritizing computational efficiency in resource-constrained devices, such as smartphones, tablets, and embedded vision applications. These architectures stand out for their computational efficiency and lightness in the size of the model. The first version of MobileNet[19] introduced depthwise separable convolutions, which reduce the number of parameters and computation compared to traditional convolutional layers. The main contribution of the MobileNetV2[20] is the inverted residual layer module with linear bottleneck *layers*. This model receives as input a compressed low-dimensional vector. The input vector is initially expanded to a larger dimensionality space, and then filters are applied to extract features. Subsequently, these features are projected back into the low-dimensional space by linear convolutions. This process is critical to the efficiency of the model, allowing valuable information to be captured in a high-dimensional space and then projected back efficiently.[20]. The MobileNetV3_small is part of the third version, MobileNetV3[21], that introduced improvements to both architecture and efficiency. One key innovation in V3 is the use of Squeeze-and-Excitation (SE) blocks that allow the model to recalibrate feature maps, improving

performance with minimal additional cost. The MobileNet version 3 comes in two variants: MobileNetV3_small and MobileNetV3_large.

The **EfficientNetV2** architecture (Tan et al., 2021) is focused on large-scale training efficiency, training speed, and model accuracy with fewer parameters. Optimization of the EfficientNetV2 model was achieved with techniques such as NAS (*Neural Architecture Search*) and parameter *scaling*. This is currently one of the most efficient architectures in terms of the use of computational resources.[22].

Table S7 shows a comparison of the number of FLOPs and the dimensionality of the features vector obtained with each model.

S.7. ML classifiers

Supervised ML classifiers LDA, KNN and SVM and RF were trained using the features vectors extracted in the previous step. Below we present briefly the learning mechanisms of these classifiers.

The LDA (Linear Discriminant Analysis) model [23] is an ML linear classification algorithm based on dimensionality reduction and the linear combination of eigenvectors for classification, using Bayes' rule. This algorithm is not directly applicable to regression problems and is suitable for classifying samples into high-dimensional spaces where instances exhibit reasonable linear separation.[24] It performs dimension reduction by transforming the original high-dimensional data into a lower-dimensional space that maximizes class separations and minimizes dispersion within classes. Test samples are sorted using Bayes' rule by considering the probabilities of each class described by a Gaussian function and that the corresponding covariances of each labeled class are equal. The decision limits created by this model are planes perpendicular to the eigenvectors obtained in dimension reduction. Considering a training dataset with n samples $[\{X_i, y_i\}]_{i=1}^{n}$, the samples are described by D-characteristics, or dimensions (high-dimensional space). It is estimated that the centroid of each class, μ_k, and the global centroid, μ, make up the *between-class matrix*, S_B. The *within-class matrix*

S_w is given by the sum of the covariances of all classes, without the normalization factor.

$$\mu_k = \frac{1}{n_k} \sum_{i \in Ck} X_i \quad \text{S8}$$

$$\mu = \sum_{k=0}^{K} \sum_{i \in Ck} \frac{1}{n_k} X_i \quad \text{S9}$$

$$S_B = \sum_{k=0}^{K} \pi_k \left(\vec{\mu}_k - \vec{\mu}\right)\left(\vec{\mu}_k - \vec{\mu}\right)^T \quad \text{S10}$$

where n_k is the number of samples of class k and π_k is the prior probability of class k given by $\pi_k = n_k / n$. The *within-class scatter* is written as.

$$S_w = \sum_{k=0}^{K} \sum_{i \in Ck} \pi_k \left(X_i - \vec{\mu}_k\right)\left(X_i - \vec{\mu}_k\right)^T \quad \text{S11}$$

Fischer's criterion for a generalized multiclass LDA is to find the optimal transformation matrix W that maximizes the objective function written below.

$$W = \arg\max_{W} \frac{\det(W^T S_B W)}{\det(W^T S_w W)} \quad \text{S12}$$

The W matrix performs the dimensional reduction of the data in the original high-dimensional space to a lower dimensional space with the maximum $K-1$ base eigenvectors, where K is the number of classes established for the problem. This optimization leads to a generalized eigenvector problem where we have to solve for maximum eigenvalues and their corresponding eigenvectors of the matrix $S_w^{-1} S_B$.

$$S_w^{-1} S_B W = \lambda W \quad \text{S13}$$

The eigenvectors that expand the "optimal" space of features and the eigenvalues represent the importance of these optimal features or dimensions to the data according to their classes or labels. The basis vectors (at most K-1) are normal to the decision planes and the instances of the data are classified among the different k-classes, depending on the region in which they project into the "optimal" space characteristics.

An equivalent way to derive the classification limits of the LDA model is by using Bayes' rule, considering the probability distributions of the samples, given the classes, being represented as a Gaussian distribution, and considering all covariances for each class k being equal. In this way, the predicted class is the one that maximizes log-posterior. We can write the log-posterior for the probability of a given sample x being classified in class k, in the case of LDA, as follows:

$$\log P(y=k \mid x) = \log \pi_k - \frac{1}{2}(x-\mu_k)\Sigma^{-1}(x-\mu_k)^T + cte \qquad \text{S14}$$

In the equation above π_k is the prior probability of class K, μ_k is the centroid of the training data of class K, and Σ^{-1} is the inverse of the covariance matrix estimated as the pooled covariance of the training data of all classes.

$$\Sigma = \frac{1}{n-K}\sum_{k=0}^{K}\sum_{i \in Ck}(X_i-\mu_k)(X_i-\mu_k)^T \qquad \text{S15}$$

In real data, the covariances for each class k are different, and if we use different covariances in Bayes' rule, we get the equation for the *Quadratic Discriminant Analysis* (QDA) model. The decision limits become curves as there are nonlinear relationships in the data.[24] The advantage of the LDA methods for the present study is that we can evaluate and compare the performance of different feature extractors with different numbers of features, since the LDA method has implicit dimension reduction before the linear classifier. Additionally, this model does not require us to optimize many hyperparameters. LDA is sensitive to large data with a small number of observations and can lead to overfitting or poorer performance.[25]

The KNN (K – Nearest Neighbors) is a non-parametric ML algorithm that performs predictions based on the number, k, of training samples of each class near the test sample. The only hyperparameter we have to choose is the number of neighbors k to account for in predictions (or a given radius). Given a test sample, the KNN model finds the k nearest neighboring matching samples among the training data and compares the number of samples of each class. The class with the highest number of observations among the k nearest neighbors of the test specimen determines the class of the test specimen. There is no training process, only the training data itself is "memorized". Distances can be calculated using any distance metric (the Euclidean metric is the most commonly used). Despite its simplicity, this algorithm has a high computational cost for large training datasets. The model can work well on a variety of issues, requires no dimension reduction, and can handle non-linear decision boundaries. However, distance-based algorithms suffer from the "*curse of dimensionality*", where effectiveness can decrease as the number of dimensions increases. It turns out that in high-dimensional spaces, the notion of distance becomes less significant. In such cases, feature selection or dimensionality reduction may be necessary to improve the accuracy of the algorithm's predictions. The KNN classifiers give results close to those of the Bayes classifier (James et al., 2023).

The SVM (Support Vector Machines) model is a supervised ML algorithm for classification and regression. This algorithm was developed by Vladmir Vapnik and collaborators based on the concepts of statistical learning theory developed by Vapnik since 1965 (Vapnik, 2000), with improvements introduced in 1992. [27] The SVM method was introduced in a general way in an article published in 1995.[28] Briefly, the idea of this algorithm is to map the training data, the so-called "support vectors", in a space of dimension larger than the original using a nonlinear transformation chosen a priori (*kernel*). In this higher-dimensional space, hyperplanes are constructed to separate the data belonging to the different labeled classes using rules that seek to generalize the problem. The method uses the set of *support-vectors* to learn the decision boundary. The SVM algorithm with linear kernel meets the decision boundaries given by equation S16 .

$$w \cdot X_i + b = 0 \quad \text{S16}$$

where w and b are parameters that minimize the objective function data by equation S17.

$$w = \underset{w}{\arg\min} \frac{|w|^2}{w} \quad \text{S17}$$

Since the objective function is quadratic and the parameters are linear, the minimization can be solved using the Lagrangian multipliers. In the case of nonlinear problems, the method uses the kernel trick on vectors X_i to map the nonlinear space into a linearly separable space. The parameters w and b the decision hypersurface are estimated from the training data. The class or label of a test specimen X_i can be predicted by the equation S18:

$$y_i = \begin{cases} 1, \, if \;\; w \cdot X_i + b > 0 \\ -1, \, if \;\; w \cdot X_i + b < 0 \end{cases} \quad \text{S18}$$

The SVM algorithm is robust in data represented in high-dimensional spaces, and the convexity of the optimization problem formulated in its training implies the existence of a single global minimum. [26] These are advantages that favor the application in this study to evaluate the quality of methods for extracting features with a high number of dimensions.

The Random Forest (RF) model is based on decision trees including the concepts of *Bagging*, *random feature selection*, random split, among others, which are applied to the construction of a set of decision trees ("*weak learners*") forming a random forest. The prediction is made by the democratic vote or by the individual prediction of each tree that makes up the RF model. This model was proposed by Leo Breiman in the article "Random forests" published in 2001.[29]

The basic idea of RF is to generate a robust model based on a set of decision trees (*ensemble*) correcting the main problems of the decision trees model. The latter is a simple model (*weak learner*), susceptible to overfitting. With the introduction of the concepts of Bagging and random selection of dimensions, the RF model decreases the bias of the decision trees. Decision trees are constructed using random subsets of the training data (*bootsraped datasets*), which consist of building a new dataset from the original dataset with the same size as the original dataset, randomly repeating some inputs (in this case with repetition). They can also be obtained without randomly repeating the data, forming a smaller dataset (in this case without substitution) and each tree is trained using a random subset of the variables or dimensions. Typically, we randomly select from each *bootstraped dataset* a number of dimensions approximately equal to the square root of the total number of dimensions (but this hyperparameter can be adjusted). The trees trained with the random subsets of data and the chosen random selection hyperparameters will be aggregated to the RF model. In this way, each RF model tree "learns" a part of the training dataset, and a part of the dimension set. This decreases tree bias, the diversity of simple classifiers increases the variance of the model, and the use of random selection of dimensions helps to reduce variance by creating more uncorrelated trees. These procedures reach a balance between bias and variance of the model. The RF model allows control of the hyperparameters related to the individual trees, the method of generating random training datasets and the voting method for the prediction made by the ensemble of trees. The greater number of tuning hyperparameters may make this model less attractive than the LDA, KNN, and SVM models. For the present study, in principle it would be necessary to adjust a large set of hyperparameters to obtain an optimized model for each feature extraction algorithm and each application. However, the RF model stands out for being very robust for presenting good performance even using the predefined hyperparameters. Another advantage is that this model has feature selection mechanisms that allow one to build good models on datasets with high dimensionality, highlighting or considering only the dimensions with the greatest contribution or greater importance.[24]

S.8. ML metrics

Multiclass accuracy

It is an extension of the accuracy of binary classification. This metric evaluates the performance of the model to classify the instances across all classes equally.

$$Multi-class\_Accuracy = \frac{correct\_predictions}{total\_predictions}$$

$$Multi-class\_Accuracy = \frac{\sum_i TP_i + TN_i}{\sum_i TP_i + TN_i + FP_i + FN_i} \qquad S19$$

For an unbalanced dataset, the value of this metric may be less representative as the classes with small numbers of instances will be neglected compared to classes with higher number of instances.

Precision – positive accuracy

This metric focuses on the instances predicted as positive out of total samples predicted as positives. It is particularly useful when the cost of False Positives is high. It is also called the accuracy of the positive predictions.

$$\Pr ecision\_i = \frac{TP_i}{TP_i + FP_i} \qquad S20$$

True positive rate – Recall

The metric Recall, also called sensitivity, is a measure of the models' ability to capture all positive instances without missing any. This metric is used to focus on the model's ability to correctly identify the positive class instances. The positive class i recall is defined as in equation S21:

$$\mathrm{Re}call\_i = \frac{TP_i}{TP_i + FN_i} \quad \text{S21}$$

For a sensor to detect SARS-CoV-2, the cost of false negatives is higher because you can leave infected people without care. On the other hand, the cost of false positives is lower as the immunosensor can be used for a quick and inexpensive test that recommends the patient to perform a more sensitive test for confirmation.

F1-Score

The F1_score represents the harmonic mean between the precision and the recall of the negative class. This metric was considered because we want the immunosensor to have not only good specificity (minimizing False Negatives) but also seeking to minimize the number of False Positives and misclassifications in the control negative tests.

$$F1\_score = \frac{2}{\frac{1}{\mathrm{Pr}ecision} + \frac{1}{\mathrm{Re}call}} = 2\frac{\mathrm{Pr}ecision \cdot \mathrm{Re}call}{\mathrm{Pr}ecision + \mathrm{Re}call} \quad \text{S22}$$

After accuracy, the metric recall is the most important as it represents the sensitivity of the immunosensor in detecting the positive SARS-CoV-2 virus. In the case of the SARS-CoV-2 immunosensor the Recall metric applies the penalty for false negatives, not considering the false positives. Although the aim is to produce a biosensor that always responds accurately and sensitively, with the minimum of false positives and false negatives, in the SARS-CoV2 test it is essential to reduce false negatives because its damage is greater than the damage of false positives.

These metrics were calculated by taking the average and standard deviations in a cross-validation. In this article we performed a stratified k-fold cross validation with k = 5, repeated 3 times with random splits. In this process the test/training splits were divided in stratified way conserving the proportion of classes in the

entire dataset in each sub dataset. The dataset is split into 5 equal parts, keeping the same proportion of samples in each split. One part is used for testing (validation) and the remaining parts are used for training. In the subsequent iteration another part is reserved for validation and the other 4 parts for training, and so on until the 5-th fold split. Then the process is repeated 3 times with random different samples in each split.

S.9. Optical Microscopy images of AuNI/glass immunosensors.

A selection of optical microscopy images of the AuNI/glass based immunosensors are as shown in **Figure S6**. It is not possible to perceive differences among the images with naked eyes. The texture characteristic changes result from adsorption of the SARS-CoV-2 virus.

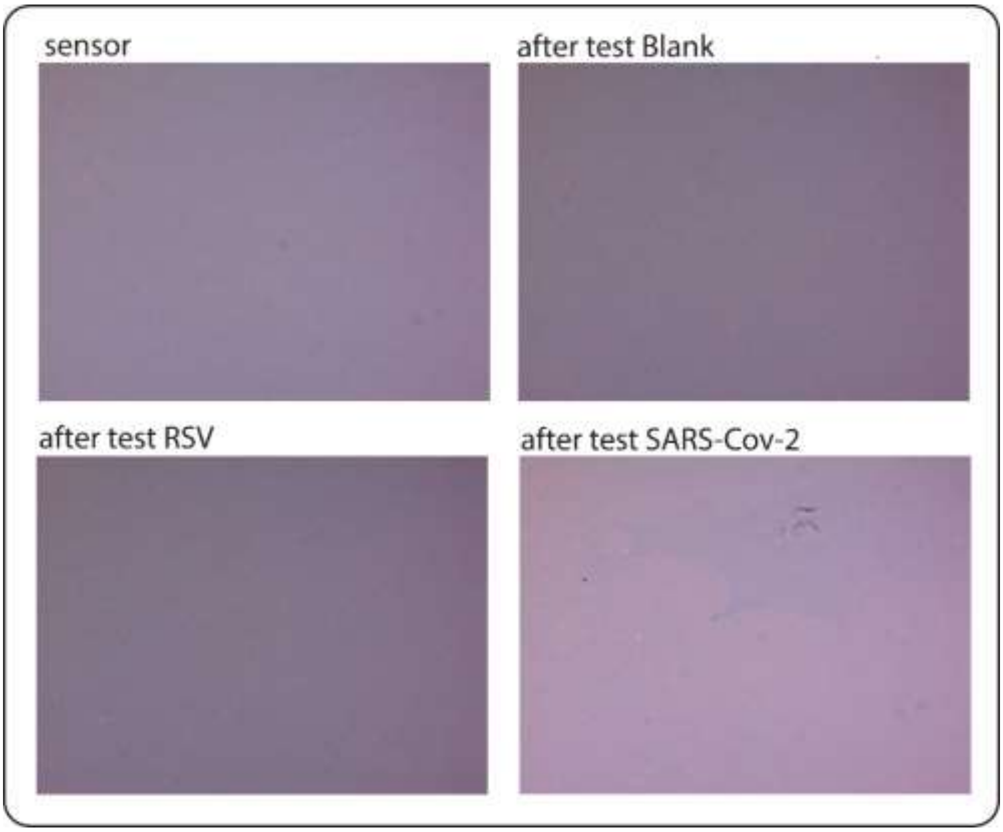

Figure S6. Images of plasmonic AuNI/glass immunosensors with optical microscopy (400X magnification approximately). We show the immunosensor before the test (sensor), after the tests with the SARS-CoV-2 virus, blank test and RSV test.

S.10. IDMAP of the predicted probabilities

The model MobileNetV3_small + SVM was trained with all samples and applied to classify these samples. The IDMAP method was used to visualize the predicted probabilities information for all samples of the dataset. The predicted probabilities for a given sample is an n-dimensional vector where n is the number of classes considered in the training. Applying IDMAP we create a 2D projection conserving the similarity of the points in the original space. The present analysis was used to visualize the model's ability to learn to distinguish the different classes. That is why the measures used in the training were also applied in the prediction. In **Figure S7** we show the IDMAP projections for the Model MobileNetV3_small_SVM trained for multiclassification of images considering different numbers of positive classes and one negative class. We discussed in the manuscript the minimum concentrations detectable via image classification. It is estimated based on the maximum of the accuracy of the model, the maximum silhouette coefficient and better class separation observed in the IDMAP projection in the scenario with 6 positive classes.

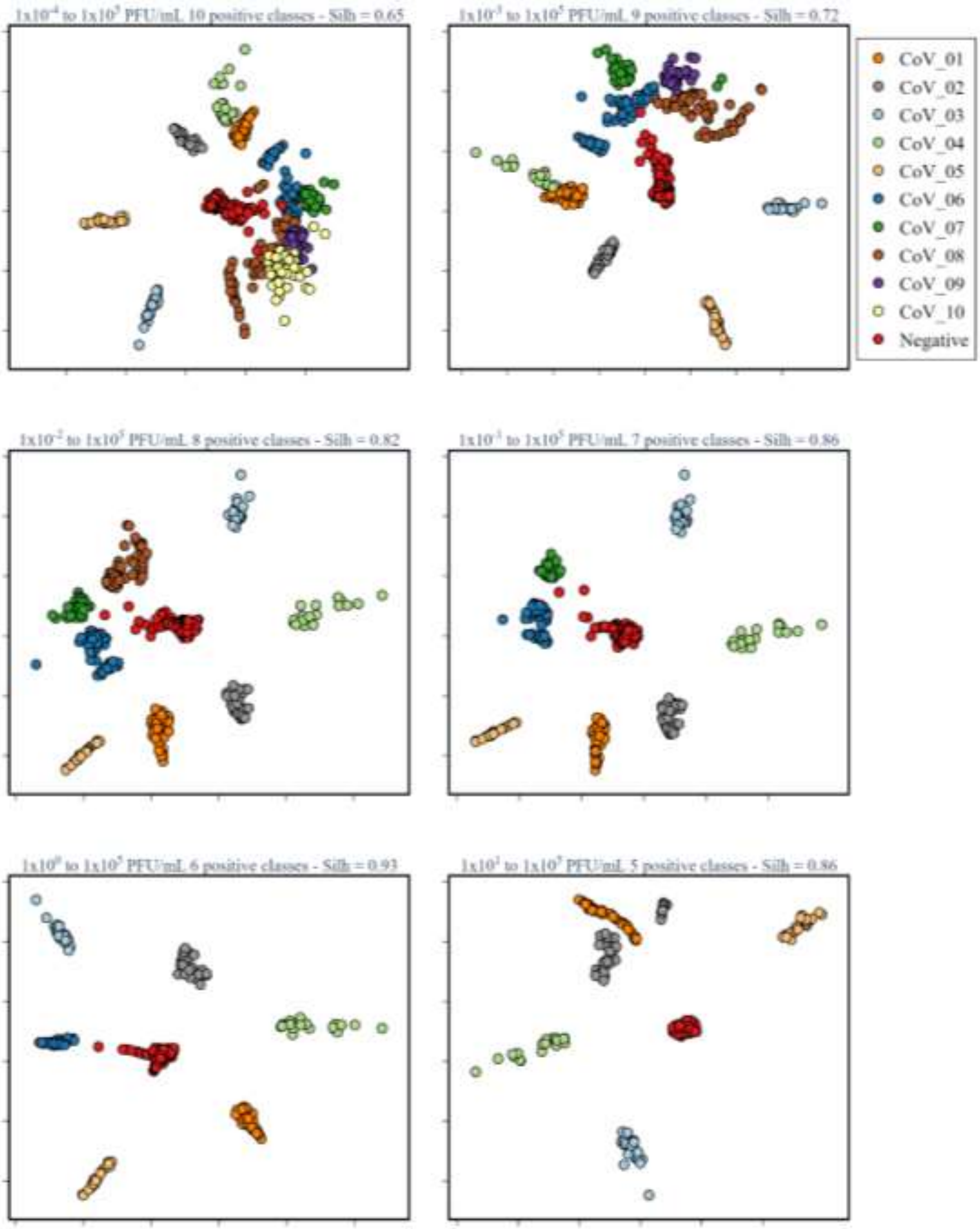

Figure S7. IDMAP of the predicted probabilities obtained from the model MobileNetV3_small + SVM for different numbers of positive classes and one negative class. The 10 positive classes represent the tests with SARS-CoV-2 with concentrations between $1x10^{-4}$ to $1x10^{5}$ PFU/mL. Removing the most diluted class $1x10^{-4}$ PFU/mL we built the multiclassification training with 9 positive classes between $1x10^{-3}$ and $1x10^{5}$ PFU/mL, and so on. The last multiclassification training was made with 5 positive classes and one negative class. The silhouette coefficients were used as a proxy of the cluster separation and coercion.

Figure S8 plots the silhouette coefficient for the IDMAP projections in Figure S7 with different numbers of positive classes.

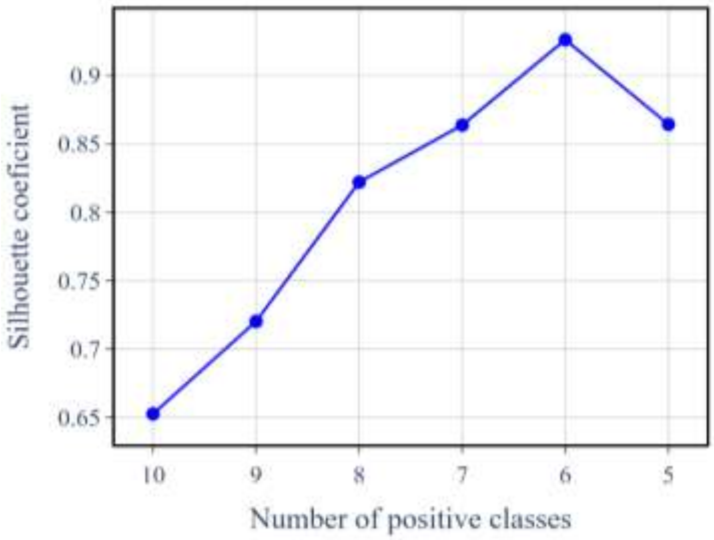

Figure S8. Silhouette coefficient of the IDMAP projections of the predicted probabilities obtained from the model MobileNetV3_small + SVM trained with one negative class and different numbers of positive classes, from 10 to 5. The highest value of the silhouette coefficient, 0.93, was obtained with 6 positive classes, corresponding to concentrations from $1x10^0$ to $1x10^5$ PFU/mL.

The handcrafted model RGB5D-LBP (P=8, R=1) + RF was trained with all samples and applied to classify these samples for different scenarios, considering different numbers of positive classes and one negative class. The predicted probabilities in each case were projected on 2D using IDMAP to visualize information for all samples. **Figure S9** shows the IDMAP projections of the predicted probabilities obtained with the model RGB5D-LBP (P=8, R=1) + RF, for different numbers of positive classes. Removing the lower concentrations classes results in more separated clusters and higher silhouette coefficients. The scenario where the best handcrafted model was trained with 6 positive classes

and one negative class resulted in an IDMAP projection with all clusters well separated, with a silhouette coefficient of 0.91.

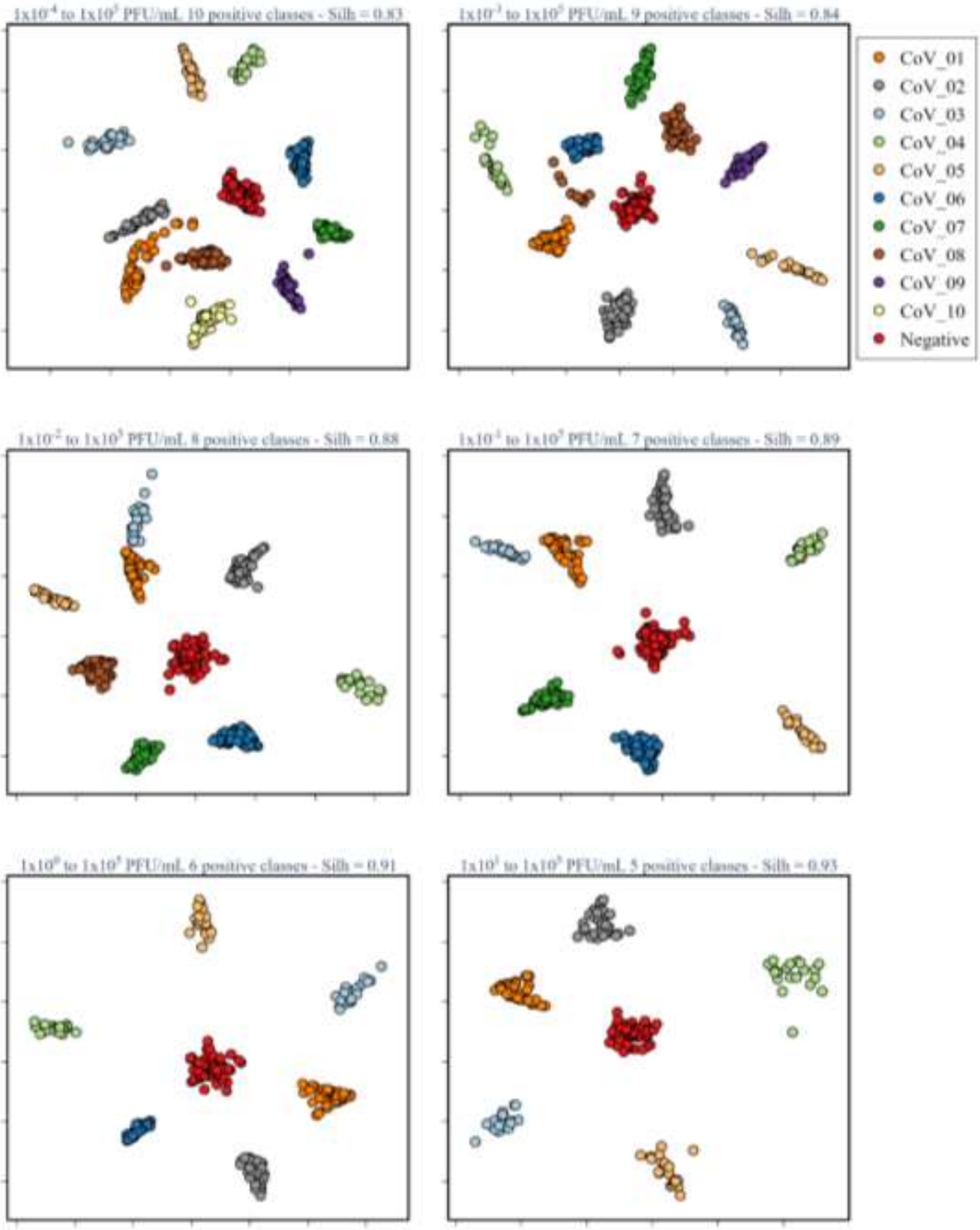

Figure S9. IDMAP of the predicted probabilities obtained from the model RGB5D-LBP + RF for different numbers of positive classes and one negative class. The 10 positive classes represent the tests with SARS-CoV-2 with concentrations between $1x10^{-4}$ and $1x10^{5}$ PFU/mL.

S.11. Results of all classification models compared.

The results and the ranking of the accuracy performance of the models based on CNN feature extraction, using RGB images are in Table S1. These results

represent the image multiclassification among 6 positive classes and one negative class, corresponding to positive SARS-CoV-2 tests with concentrations from $1x10^0$ to $1x10^5$ PFU/mL and the negative class corresponding to tests Probe, Blank and RSV. The metrics Accuracy, F1-score of the negative class and the average of the F1-scores of the positive classes were computed during cross-validation with stratified k-fold, k=5, repeated 3 times. The ranking represents the order of the higher accurate models considering the 48 CNN based models and the 44 Handcrafted models compared. The model **MobileNetV3_small + SVM** demonstrated the best performance with the **accuracy score of 91.6 ± 2.2 %.**

Table S1. Evaluation results for the multiclassification problem with 6 positive classes and one negative class, corresponding to positive SARS-CoV-2 tests with concentrations from 1 to $1x10^5$ PFU/mL, for 48 models formed by CNN feature extractors with the LDA, KNN, SVM and RF classifiers, using the original RGB images in the input. The metrics Accuracy, F1-score of the negative class and the average of the F1-scores of the positive classes were computed during cross-validation. The ranking represents the order of the higher accurate models considering all CNN based and the 44 Handcrafted based models compared. We highlight in yellow the MobileNetV3_small + SVM model that showed best performance.

ind	Ranking	Feature extractor	Classifier	Accuracy	F1 Neg class	Avg F1 pos classs
1	**1**	**MobileNetV3_small**	**SVM**	**91.6 ±2.2**	96.9 ±1.3	78.8 ±17.9
2	2	DenseNet121	SVM	91.0 ±2.5	96.8 ±1.3	76.9 ±17.1
3	3	ResNet34	SVM	90.0 ±2.1	96.1 ±1.2	76.2 ±20.8
4	4	MobileNetV3_small	LDA	89.9 ±1.7	96.6 ±0.9	72.5 ±27.6
5	5	ResNet18	SVM	89.9 ±2.7	96.4 ±1.3	71.6 ±25.0
6	6	EfficientNetV2_S	SVM	89.6 ±2.9	96.0 ±1.5	74.2 ±18.9
7	7	EfficientNetV2_B1	SVM	89.3 ±2.7	95.5 ±1.8	74.9 ±20.1
8	8	DenseNet121	LDA	89.0 ±2.3	95.6 ±1.3	70.9 ±25.6
9	9	VGG19	SVM	89.0 ±1.8	96.3 ±1.0	69.6 ±20.5
10	10	EfficientNetV2_B0	SVM	88.8 ±3.2	95.5 ±1.9	74.3 ±15.0
11	11	EfficientNetV2_S	LDA	88.3 ±2.7	95.1 ±1.7	70.9 ±26.1
12	12	MobileNetV3_small	RF	88.1 ±2.7	94.3 ±1.8	65.2 ±30.6
13	13	ResNet18	LDA	87.8 ±2.1	95.1 ±1.2	67.2 ±17.1
14	14	EfficientNetV2_B1	LDA	87.7 ±2.6	94.3 ±1.3	69.6 ±29.2
15	15	EfficientNetV2_M	SVM	87.7 ±3.0	94.8 ±1.4	72.0 ±22.6
16	16	ResNet18	RF	87.3 ±2.4	93.5 ±1.3	59.4 ±26.3
17	17	VGG16	SVM	87.1 ±2.2	95.4 ±1.0	64.6 ±20.6
18	18	EfficientNetV2_B0	LDA	86.8 ±2.2	94.3 ±1.3	66.7 ±24.6
19	19	EfficientNetV2_B1	RF	86.5 ±2.8	93.6 ±1.7	58.8 ±27.3
20	20	EfficientNetV2_M	LDA	86.4 ±2.2	93.5 ±1.3	68.5 ±23.9
21	21	ResNet34	LDA	86.3 ±2.6	94.9 ±1.5	63.6 ±32.6
22	22	VGG19	LDA	86.2 ±3.9	94.4 ±2.4	60.4 ±24.8
23	23	MobileNet	SVM	86.0 ±2.7	94.0 ±1.5	66.3 ±25.6
24	24	DenseNet121	RF	85.9 ±1.6	93.1 ±1.2	54.3 ±27.2
25	25	VGG19	RF	85.0 ±2.1	92.8 ±1.5	48.0 ±25.6

26	26	ResNet34	RF	84.9 ±2.2	92.3 ±1.5	52.9 ±29.3
27	27	EfficientNetV2_B0	RF	84.8 ±2.6	92.9 ±1.3	51.6 ±25.7
28	28	EfficientNetV2_S	RF	84.7 ±2.8	92.6 ±1.7	53.6 ±22.7
29	29	EfficientNetV2_S	KNN	84.6 ±3.2	93.7 ±1.9	60.1 ±23.8
30	31	ResNet18	KNN	83.9 ±2.5	94.0 ±1.5	57.0 ±27.4
31	32	MobileNetV3_small	KNN	83.9 ±2.5	94.3 ±1.3	53.9 ±25.3
32	33	ResNet34	KNN	83.7 ±2.8	93.2 ±1.6	59.2 ±24.1
33	34	VGG16	RF	83.7 ±2.3	91.5 ±1.8	44.4 ±19.8
34	35	MobileNet	LDA	83.6 ±2.7	92.1 ±1.7	58.6 ±31.4
35	36	VGG19	KNN	83.6 ±2.8	94.5 ±1.7	52.0 ±21.3
36	37	EfficientNetV2_M	KNN	83.4 ±2.5	93.9 ±1.6	55.4 ±27.6
37	39	VGG16	KNN	83.1 ±2.8	94.8 ±2.2	51.8 ±19.9
38	40	MobileNet	RF	83.1 ±2.2	91.3 ±1.0	47.1 ±22.1
39	41	EfficientNetV2_M	RF	83.1 ±2.3	91.7 ±1.5	52.0 ±27.0
40	42	VGG16	LDA	83.1 ±3.1	92.9 ±2.3	54.0 ±25.5
41	46	MobileNetV2	SVM	82.7 ±3.0	92.5 ±1.9	57.7 ±24.3
42	47	DenseNet121	KNN	82.6 ±2.5	93.4 ±1.8	52.6 ±27.6
43	48	EfficientNetV2_B1	KNN	82.2 ±2.8	93.3 ±1.7	49.9 ±20.7
44	50	EfficientNetV2_B0	KNN	82.0 ±2.8	93.1 ±2.1	49.6 ±20.7
45	53	MobileNetV2	LDA	81.8 ±1.9	91.2 ±1.4	51.8 ±24.8
46	54	MobileNetV2	RF	81.5 ±2.7	90.2 ±1.7	34.0 ±19.8
47	58	MobileNet	KNN	81.0 ±2.3	93.5 ±1.2	47.8 ±23.9
48	62	MobileNetV2	KNN	79.5 ±3.4	92.1 ±1.9	43.8 ±20.5

We compared 44 handcrafted models as shown in Table S2. The metrics Accuracy, F1-score of the negative class and the average of the F1-scores of the positive classes were computed during cross-validation with stratified k-fold, k=5, repeated 3 times. The ranking represents the order of the higher accurate models considering all 44 Handcrafted based and 48 CNN-based models. The model **RGB5D-LBP + RF** demonstrated the best performance among the handcrafted models with the **accuracy score of 84.1 ± 2.3 %,** the F1-score of the negative class of 91.4 ± 1.4 %, with the **30th best performance** ranking among the 92 models. This method was designed in this work to extract features from the original RGB images extracting 5 components: 3 classic LBP components and 2 cross-channel square template LBP components, resulting in 50 features (with R=1, P=8), for each image (see Figure S5). The IDMAP projections of the predicted probabilities obtained for the model RGB5D-LBP_81 + RF trained with different numbers of positive classes and one negative class are shown in Figure S9.

Table S2. Evaluation of the 44 Handcrafted models for the multiclassification of 6 positive classes and one negative class, corresponding to positive SARS-CoV-2 tests with concentrations from 1x100 to 1x105 PFU/mL. The metrics Accuracy, F1-score of the negative class and the average of the F1-scores of the positive classes were computed during cross-validation. The ranking represents the order of the higher accurate models considering all Handcrafted-based and CNN-based models. The model RGB5D-LBP + RF demonstrated the best performance among the handcrafted models, ranking 29th among all models.

ind	Ranking	Feature extractor	Classifier	Accuracy	F1 Neg class	Avg F1 pos classs
1	**30**	**RGB5D_LBP_81**	**RF**	**84.1 ±2.3**	91.4 ±1.4	49.6 ±25.6
2	38	CLBP_81_smc	SVM	83.1 ±2.6	91.3 ±2.2	62.5 ±21.8
3	43	RGB5D_LBP_122	RF	83.1 ±2.3	90.7 ±1.8	46.2 ±26.9
4	44	RGB5D_LBP_163	RF	82.9 ±3.4	90.6 ±2.1	46.3 ±25.5
5	45	CLBP_81_smc	RF	82.7 ±2.5	90.6 ±1.6	47.3 ±28.5
6	49	RGB5D_LBP_81	KNN	82.1 ±3.3	91.8 ±1.9	53.4 ±19.5
7	51	GLCM	KNN	82.0 ±2.5	92.1 ±1.7	55.4 ±21.3
8	52	GLCM	RF	82.0 ±2.4	91.7 ±1.5	53.3 ±24.6
9	55	CLBP_162_smc	RF	81.5 ±2.6	90.0 ±1.6	40.3 ±30.3
10	56	CLBP_162_smc	SVM	81.3 ±2.4	91.2 ±1.4	54.4 ±24.7
11	57	CLBP_162_smc	KNN	81.2 ±3.4	91.1 ±2.0	52.1 ±22.5
12	59	CLBP_81_smc	KNN	80.7 ±3.7	89.8 ±2.1	55.0 ±26.7
13	60	RGB5D_LBP_122	KNN	80.4 ±2.8	90.2 ±1.9	49.0 ±20.6
14	61	RGB5D_LBP_163	KNN	79.8 ±2.1	89.8 ±2.0	46.4 ±24.0
15	63	CLBP_81_smc	LDA	79.2 ±2.5	88.3 ±1.7	47.9 ±18.8
16	64	CLBP_162_smc	LDA	78.5 ±2.4	88.9 ±1.5	43.1 ±25.1
17	65	LBP_81	RF	77.5 ±2.3	87.0 ±1.4	41.3 ±28.3
18	66	CLBP_81_s_mc	RF	77.2 ±2.8	86.9 ±1.8	32.0 ±21.7
19	67	LBP_81	KNN	76.5 ±2.7	87.3 ±1.6	44.0 ±22.6
20	68	GLDM	RF	74.5 ±2.6	85.9 ±1.7	31.1 ±24.5
21	69	LBP_162	RF	74.3 ±2.4	85.7 ±1.4	27.9 ±20.7
22	70	CLBP_162_s_mc	RF	74.3 ±2.4	85.5 ±1.1	22.4 ±20.2
23	71	CLBP_81_s_mc	KNN	74.3 ±2.5	85.6 ±1.6	39.0 ±20.0
24	72	CLBP_81_s_mc	LDA	73.1 ±1.9	84.6 ±1.4	24.6 ±28.7
25	73	GLDM	KNN	72.7 ±3.3	86.6 ±2.5	32.4 ±21.9
26	74	LBP_81	LDA	72.5 ±1.7	84.6 ±1.2	12.5 ±13.3
27	75	GLCM	LDA	72.1 ±2.0	83.6 ±1.4	15.3 ±12.1
28	76	GLDM	LDA	71.4 ±1.7	84.1 ±1.1	11.0 ±12.8
29	77	LBP_162	KNN	71.3 ±2.9	85.2 ±1.7	27.6 ±17.8
30	78	LBP_162	LDA	71.2 ±1.5	83.8 ±1.2	9.6 ±18.8
31	79	RGB5D_LBP_81	LDA	70.8 ±1.9	83.7 ±1.3	18.6 ±17.7
32	80	RGB5D_LBP_163	LDA	70.6 ±1.8	83.4 ±1.1	15.5 ±17.0
33	81	CLBP_162_s_mc	LDA	70.3 ±1.9	84.0 ±1.4	10.9 ±14.2
34	82	RGB5D_LBP_122	LDA	70.1 ±1.7	83.2 ±0.9	16.0 ±19.1
35	83	CLBP_162_s_mc	KNN	69.6 ±2.8	83.1 ±1.6	22.5 ±17.5
36	84	RGB5D_LBP_163	SVM	69.5 ±4.0	79.5 ±3.8	54.5 ±17.4
37	85	RGB5D_LBP_81	SVM	68.4 ±3.3	77.8 ±3.3	55.8 ±14.3
38	86	RGB5D_LBP_122	SVM	67.8 ±3.5	78.7 ±4.3	50.8 ±16.7
39	87	CLBP_81_s_mc	SVM	59.4 ±4.1	67.9 ±5.0	49.3 ±16.6
40	88	CLBP_162_s_mc	SVM	57.3 ±4.2	67.6 ±4.6	43.6 ±15.4

41	89	GLCM	SVM	53.6 ±4.1	63.3 ±5.8	44.9 ±17.5
42	90	GLDM	SVM	49.3 ±3.3	60.4 ±4.5	37.8 ±15.1
43	91	LBP_81	SVM	45.4 ±3.3	50.2 ±4.0	41.8 ±19.0
44	92	LBP_162	SVM	43.9 ±2.9	54.3 ±5.0	33.1 ±18.0

Figure S10 shows three plots for the accuracy scores, the average of F1-scores of the positive classes and the F1-score of the negative class for the scenarios with different numbers of positive classes. The accuracy score increases and reaches a maximum for 6 and 5 positive classes. The average F1-score of the positive classes also increases by removing the lower concentrations. As already discussed, the F1-score of the negative class is maintained at around 95 to 98 %. The levelling off in the plots of accuracy and average F1-scores of positive classes suggests that the image classification method loses classification ability when including all concentrations. Good accuracy is obtained to classify among 6 or 5 positive classes with the highest concentrations.

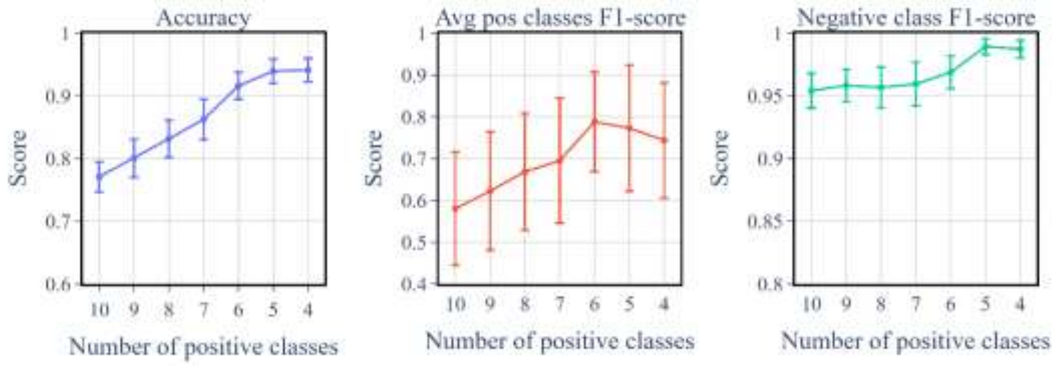

Figure S10. Multiclassification scores considering a decreasing number of positive test concentrations from 10 dilutions (all tests) to 4 positive test concentrations, by removing the most diluted positive classes from the training. After removing the 4 most diluted positive classes and training the best model with 6 positive classes and 1 negative class we obtained highest possible classification accuracies. The average of the F1-scores of the positive classes is maximum and the F1-score of the negative class did not vary considerably for the multiclassification scenarios.

The results obtained for multiclassification cannot be interpreted as yielding an LOD defined by IUPAC. It must be interpreted as the ability of the model to distinguish the images, thus functioning as a detection method. In a subsidiary experiment we trained the image classification models for binary classification of the SARS-CoV-2 tests. The concentrations considered were from 1 to $1x10^5$ PFU/mL in the class positive and the control tests (Blank, RSV and Probe tests)

in the negative class. The models were evaluated in cross-validation with stratified splits, k=5 and 3 repeats. The metrics Accuracy, Recall and Negative Predictive Value were calculated, including their average and standard deviation. The results are shown in Table S3 for the CNN models and in Table S4 for the handcrafted models. The models were ranked by the accuracy score. The best model was the **MobileNetV3_small + LDA** with the **accuracy of 96.5 ± 1.6 %**. The **model RGB5D-LBP + RF** demonstrated the best performance among the handcrafted models, with the **accuracy of 90.8 ± 1.9 %**, taking the **29th position** in the general ranking of the 92 models. The models with highest performances are highlighted in yellow.

Table S3. Results obtained with the CNN-based models for the binary detection with the SARS-CoV-2 with dilutions from 1 to 1x10^5 PFU/mL.

ind	Ranking	Feature extractor	Classifier	Accuracy	Recall	Neg Pred Val
1	**1**	**MobileNetV3_small**	**LDA**	**96.5% ±1.6**	92.6% ±4.1	96.9% ±1.7
2	2	DenseNet121	SVM	95.5% ±1.4	92.9% ±3.5	97.0% ±1.4
3	3	MobileNetV3_small	SVM	95.0% ±2.3	92.6% ±4.5	96.9% ±1.9
4	89	VGG19	LDA	94.6% ±2.2	90.0% ±4.8	95.9% ±1.9
5	4	DenseNet121	LDA	94.5% ±1.4	88.5% ±4.8	95.3% ±1.8
6	90	VGG19	RF	94.3% ±2.2	90.4% ±6.5	96.0% ±2.6
7	91	VGG19	SVM	94.1% ±1.9	89.0% ±4.3	95.4% ±1.7
8	5	EfficientNetV2_B0	SVM	93.8% ±2.2	90.2% ±3.8	95.9% ±1.6
9	6	ResNet18	SVM	93.8% ±2.2	89.3% ±6.0	95.5% ±2.4
10	7	ResNet18	LDA	93.5% ±1.9	90.4% ±5.0	95.9% ±2.0
11	8	EfficientNetV2_S	LDA	93.4% ±1.9	86.3% ±7.2	94.4% ±2.8
12	9	EfficientNetV2_B0	LDA	93.3% ±2.4	85.1% ±6.3	94.0% ±2.4
13	10	ResNet34	LDA	93.1% ±2.5	89.6% ±7.8	95.7% ±3.0
14	11	MobileNetV3_small	RF	93.0% ±2.2	86.1% ±6.7	94.3% ±2.5
15	12	ResNet18	RF	92.9% ±3.3	87.8% ±9.0	95.0% ±3.5
16	85	VGG16	LDA	92.9% ±2.1	88.3% ±6.5	95.1% ±2.6
17	13	EfficientNetV2_B1	RF	92.8% ±2.7	86.9% ±6.9	94.6% ±2.7
18	14	DenseNet121	RF	92.8% ±1.9	85.2% ±7.8	94.0% ±2.9
19	15	EfficientNetV2_S	RF	92.7% ±2.4	83.6% ±6.6	93.4% ±2.5
20	16	EfficientNetV2_B0	RF	92.6% ±2.7	85.8% ±7.4	94.2% ±2.8
21	92	VGG19	KNN	92.6% ±2.4	89.9% ±5.9	95.7% ±2.4
22	86	VGG16	SVM	92.6% ±2.1	87.5% ±5.9	94.8% ±2.3
23	17	ResNet34	RF	92.4% ±3.3	84.4% ±9.4	93.7% ±3.5
24	18	EfficientNetV2_B1	LDA	92.4% ±2.5	87.3% ±6.6	94.8% ±2.5
25	87	VGG16	KNN	92.2% ±2.5	89.9% ±5.9	95.7% ±2.4
26	19	EfficientNetV2_M	RF	92.0% ±3.1	82.3% ±8.2	92.9% ±3.2
27	20	EfficientNetV2_S	SVM	92.0% ±2.4	87.5% ±7.0	94.8% ±2.8

28	21	EfficientNetV2_M	KNN	91.8% ±2.8	90.1% ±6.0	95.7% ±2.5
29	22	MobileNetV3_small	KNN	91.6% ±2.4	87.7% ±5.3	94.8% ±2.1
30	23	EfficientNetV2_B1	SVM	91.5% ±2.9	86.9% ±6.3	94.5% ±2.5
31	24	ResNet34	SVM	91.5% ±1.8	87.0% ±5.5	94.5% ±2.2
32	25	MobileNet	KNN	91.4% ±2.0	89.7% ±5.3	95.6% ±2.2
33	26	ResNet18	KNN	91.2% ±2.2	86.9% ±5.6	94.5% ±2.2
34	27	EfficientNetV2_B0	KNN	91.2% ±2.0	86.2% ±4.9	94.2% ±1.9
35	28	MobileNet	RF	91.1% ±2.5	82.2% ±7.5	92.7% ±2.7
36	88	VGG16	RF	90.8% ±2.8	83.7% ±6.8	93.3% ±2.6
37	30	EfficientNetV2_S	KNN	90.8% ±2.7	86.2% ±6.8	94.2% ±2.7
38	31	EfficientNetV2_B1	KNN	90.4% ±2.3	86.7% ±6.8	94.4% ±2.6
39	32	DenseNet121	KNN	90.3% ±2.7	86.4% ±7.5	94.2% ±2.9
40	33	EfficientNetV2_M	LDA	90.2% ±2.7	79.8% ±8.5	91.9% ±3.2
41	34	MobileNet	LDA	90.1% ±2.6	81.8% ±5.7	92.5% ±2.2
42	35	EfficientNetV2_M	SVM	90.1% ±2.0	84.2% ±5.5	93.3% ±2.1
43	36	MobileNetV2	RF	90.0% ±2.7	81.1% ±7.1	92.2% ±2.7
44	37	ResNet34	KNN	89.9% ±2.4	84.8% ±5.9	93.6% ±2.3
45	38	MobileNet	SVM	89.8% ±2.5	81.4% ±6.9	92.3% ±2.7
46	39	MobileNetV2	LDA	89.7% ±2.3	83.3% ±5.6	93.0% ±2.2
47	46	MobileNetV2	SVM	88.6% ±2.2	81.8% ±5.9	92.4% ±2.3
48	47	MobileNetV2	KNN	88.4% ±2.6	85.6% ±6.9	93.8% ±2.8

Table S4. Results obtained with the Handcrafted models for the binary detection with the SARS-CoV-2 with dilutions from 1 to $1x10^5$ PFU/mL.

ind	ranking	Feature extractor	Classifier	Accuracy	Recall	Neg Pred Val
1	**29**	**RGB5D_LBP_81**	**RF**	**90.8% ±2.0**	79.9% ±7.1	91.9% ±2.5
2	40	CLBP_81_smc	RF	89.5% ±2.2	75.7% ±6.0	90.3% ±2.2
3	41	GLCM	RF	89.1% ±3.2	80.3% ±7.8	91.9% ±3.0
4	42	GLCM	KNN	88.9% ±2.8	83.1% ±8.1	92.9% ±3.2
5	43	CLBP_162_smc	RF	88.9% ±2.7	77.1% ±5.6	90.7% ±2.2
6	44	RGB5D_LBP_122	RF	88.7% ±1.9	74.9% ±8.1	90.0% ±2.8
7	45	RGB5D_LBP_163	RF	88.7% ±1.1	75.7% ±6.8	90.3% ±2.3
8	48	RGB5D_LBP_81	KNN	88.3% ±2.6	84.8% ±5.4	93.4% ±2.2
9	49	CLBP_162_smc	KNN	87.8% ±3.6	82.3% ±7.5	92.4% ±3.0
10	50	RGB5D_LBP_122	KNN	86.4% ±2.6	79.6% ±7.3	91.3% ±2.6
11	51	CLBP_81_smc	KNN	85.9% ±2.8	82.2% ±6.0	92.2% ±2.4
12	52	RGB5D_LBP_163	KNN	85.7% ±2.2	76.1% ±7.9	90.0% ±2.8
13	53	RGB5D_LBP_81	SVM	83.5% ±2.6	92.3% ±4.4	96.1% ±2.1
14	54	CLBP_81_smc	SVM	82.7% ±3.8	83.7% ±7.7	92.4% ±3.3
15	55	CLBP_81_s_mc	RF	81.4% ±3.0	61.8% ±8.1	84.9% ±2.8
16	56	LBP_81	RF	80.9% ±3.8	58.6% ±5.2	83.8% ±2.2
17	57	LBP_81	KNN	80.8% ±3.8	61.1% ±7.4	84.5% ±2.7
18	58	CLBP_162_smc	LDA	80.6% ±3.1	60.6% ±7.1	84.4% ±2.4
19	59	CLBP_162_smc	SVM	80.1% ±2.9	66.3% ±9.0	86.0% ±3.1

20	60	GLDM	KNN	80.0% ±2.4	59.9% ±8.2	84.1% ±2.6
21	61	GLDM	RF	79.9% ±4.5	57.1% ±11.3	83.3% ±3.7
22	62	RGB5D_LBP_122	SVM	79.5% ±3.2	94.8% ±3.5	97.1% ±1.9
23	63	CLBP_162_s_mc	RF	79.1% ±2.7	54.5% ±7.9	82.4% ±2.4
24	64	LBP_162	KNN	79.1% ±2.4	60.7% ±7.9	84.1% ±2.5
25	65	RGB5D_LBP_81	LDA	79.1% ±2.2	50.9% ±6.6	81.5% ±1.8
26	66	CLBP_81_smc	LDA	79.0% ±2.7	53.7% ±10.2	82.3% ±3.0
27	67	CLBP_81_s_mc	KNN	78.9% ±2.0	65.1% ±6.4	85.3% ±2.2
28	68	RGB5D_LBP_122	LDA	78.6% ±2.3	53.7% ±6.2	82.1% ±1.8
29	69	RGB5D_LBP_163	LDA	78.5% ±2.2	49.8% ±6.9	81.1% ±1.9
30	70	LBP_162	RF	77.9% ±3.0	54.7% ±7.0	82.2% ±2.2
31	71	CLBP_162_s_mc	LDA	76.2% ±2.1	40.1% ±6.6	78.4% ±1.8
32	72	GLCM	LDA	75.8% ±1.7	29.9% ±6.5	76.4% ±1.5
33	73	RGB5D_LBP_163	SVM	75.7% ±3.0	93.2% ±4.5	96.1% ±2.4
34	74	LBP_162	LDA	75.6% ±2.4	35.1% ±6.9	77.3% ±1.9
35	75	CLBP_162_s_mc	SVM	75.3% ±2.3	87.2% ±6.5	93.1% ±3.1
36	76	GLDM	LDA	74.8% ±2.1	32.2% ±5.6	76.5% ±1.4
37	77	LBP_81	LDA	74.7% ±1.7	37.8% ±6.5	77.5% ±1.6
38	78	CLBP_81_s_mc	SVM	74.7% ±2.8	85.8% ±7.0	92.3% ±3.4
39	79	CLBP_81_s_mc	LDA	73.8% ±2.2	34.9% ±6.1	76.7% ±1.6
40	80	CLBP_162_s_mc	KNN	73.5% ±3.0	48.1% ±8.8	79.5% ±2.8
41	81	GLDM	SVM	72.1% ±3.8	81.5% ±4.5	89.7% ±2.4
42	82	LBP_162	SVM	70.8% ±3.5	73.9% ±6.0	86.4% ±2.9
43	83	GLCM	SVM	70.3% ±2.4	70.1% ±4.8	84.9% ±1.9
44	84	LBP_81	SVM	69.2% ±3.9	75.5% ±5.8	86.6% ±2.9

S.12. RGB vs grayscale images: a comparison.

The CNN models can be trained using RGB images or images converted to grayscale. We compared the performance of the CNN models using RGB images or grayscale images as inputs. A total of 12 CNN-based feature extractors were analyzed, with the four classifiers (LDA, KNN, SVM and RF) corresponding to a total of 48 models. The performance of most of these CNN-based models outperformed those using handcrafted feature extractors. The handcrafted feature extraction methods LBP, CLBP, GLCM and GLDM are applied only to grayscale images, while the method RGB5D-LBP was developed to extract texture features from RGB images. The best performance among the handcrafted models was obtained with the model RGB5D-LBP + RF. This performance can be attributed to the optimized feature extraction algorithm that considers both the grayscale and the RGB color textures in the immunosensor

images. To compare the performance of the CNN models with RGB and grayscale images we considered the sum of the differences of the accuracy scores of the models using RGB and grayscale images (RGBvsGray_score), according to Equation S23. For a given CNN model i, if $Acc_i^{RGB} - Acc_i^{gray}$ is positive, the CNN model performed better using RGB images compared to using grayscale images as inputs.

$$RGBvsGray\_scorer = \frac{1}{CNN\,\mathrm{mod}\,els} \sum_{i}^{CNN\mathrm{mod}\,els} (Acc_i^{RGB} - Acc_i^{gray}) \qquad \text{S23}$$

The 48 CNN models analysed demonstrated positive value for $Acc_i^{RGB} - Acc_i^{gray}$ (Table S5). The models were trained for the multiclassification of the SARS-CoV-2 concentrations between $1x10^0$ to $1x10^5$ PFU/mL (6 positive classes) and 1 negative class. The average RGBvsGray_score is 3.2 %, which is close to the error in the accuracies calculated in the cross-validation. The small improvement in performance of the CNN models using RGB images compared to the models using grayscale suggests that the color information is relevant for the classification but the major contributor for detection of the SARS-CoV-2 virus in the plasmonic immunosensor is in the grayscale.

Table S5. RGB vs grayscale comparison of CNN models. Difference of accuracies of the CNN models using RGB images and grayscale images as inputs. The CNN models performed better using RGB images.

ind	Feature Extractor	Classifier	RGBvsGray_score
1	MobileNetV3_small	SVM	4.6 ±2.5
2	DenseNet121	SVM	4.0 ±2.8
3	ResNet34	SVM	3.2 ±2.4
4	MobileNetV3_small	LDA	3.1 ±3.1
5	ResNet18	SVM	3.2 ±3.7
6	EfficientNetV2_S	SVM	3.1 ±2.9
7	EfficientNetV2_B1	SVM	3.3 ±2.7
8	DenseNet121	LDA	3.8 ±2.4
9	VGG19	SVM	4.0 ±2.7
10	EfficientNetV2_B0	SVM	4.3 ±3.2
11	EfficientNetV2_S	LDA	3.9 ±2.7
12	MobileNetV3_small	RF	3.7 ±3.4

13	ResNet18	LDA	3.4 ±2.2
14	EfficientNetV2_B1	LDA	3.5 ±2.6
15	EfficientNetV2_M	SVM	3.5 ±3.0
16	ResNet18	RF	3.4 ±2.4
17	VGG16	SVM	3.4 ±2.2
18	EfficientNetV2_B0	LDA	3.1 ±2.6
19	EfficientNetV2_B1	RF	2.9 ±2.8
20	EfficientNetV2_M	LDA	2.9 ±3.9
21	ResNet34	LDA	3.5 ±2.8
22	VGG19	LDA	3.5 ±3.9
23	MobileNet	SVM	3.3 ±2.7
24	DenseNet121	RF	3.3 ±1.9
25	VGG19	RF	3.0 ±3.4
26	ResNet34	RF	2.9 ±2.3
27	EfficientNetV2_B0	RF	3.4 ±2.6
28	EfficientNetV2_S	RF	3.4 ±2.8
29	EfficientNetV2_S	KNN	3.3 ±3.2
30	ResNet18	KNN	2.7 ±3.1
31	MobileNetV3_small	KNN	2.8 ±3.0
32	ResNet34	KNN	2.6 ±2.8
33	VGG16	RF	2.6 ±2.8
34	MobileNet	LDA	2.8 ±2.7
35	VGG19	KNN	2.8 ±3.1
36	EfficientNetV2_M	KNN	2.7 ±2.5
37	VGG16	KNN	2.4 ±2.8
38	MobileNet	RF	2.5 ±3.0
39	EfficientNetV2_M	RF	2.6 ±3.0
40	VGG16	LDA	2.6 ±3.1
41	MobileNetV2	SVM	2.5 ±3.0
42	DenseNet121	KNN	2.5 ±2.5
43	EfficientNetV2_B1	KNN	2.9 ±3.7
44	EfficientNetV2_B0	KNN	2.8 ±2.8
45	MobileNetV2	LDA	3.3 ±2.6
46	MobileNetV2	RF	3.3 ±2.9
47	MobileNet	KNN	4.1 ±2.9
48	MobileNetV2	KNN	2.8 ±3.4

S.13. Processing time for the feature extraction methods

Table S6 compares the number of features extracted from the image and the execution time (s) of the handcrafted algorithms used. These times were obtained for original images with resolution (2560x1920x3) applied to each method

considering the conversion to grayscale, if required by the method, and using a laptop with Intel(R) core™ i7-6700HQ CPU with four 2.6GHz physical cores, 16GB of RAM and NVIDIA GeForce GTX960M graphics acceleration card (GPU) with 2GB of dedicated memory. The GPU card was not used.

Table S6. Comparison between the dimensionality of the feature vector and execution times of the handcrafted extraction algorithms.

Feature extractor	n_features	sec_iteraction
GLCM	16	0.3
GLDM	16	0.9
LBP_81_riu2	10	1.7
LBP_162_riu2	18	3.8
CLBP_81_s_mc	30	3.7
CLBP_81_s/m/c	200	3.8
CLBP_162_s_mc	54	6.6
CLBP_162_s/m/c	648	6.7
RGB5D-LBP_81	54	9.3
RGB5D-LBP _122	70	13.8
RGB5D-LBP _163	90	18.0

In Table S7 we compare the number of features extracted, the number of FLOPs ("Floating Point Operations") and processing time (s) of yjr CNN-based feature extractors employed. These execution times were achieved for each image in the original RGB (2560x1920x3) resolution applied to each method considering the conversion to grayscale and using the computing resources provided for free by Google Colab[30] with an Intel(R) Xeon(R) CPU with two cores of @2.20GHz, 13 GB of RAM, disk space of 78.2 GB, and an NVIDIA Tesla T4 GPU with dedicated memory of 16GB (VRAM).

Table S7. Comparison between the dimensionality of the feature vector, number of FLOPs and execution times of CNN-based feature extractors.

Feature extractor	FLOPs	n_features	sec_iteraction
MobileNetV3_small	0.6	576	0.18
DenseNet121	29.8	1024	0.64
ResNet34	19.2	512	0.29
ResNet18	9.5	512	0.24
EfficientNetV2_S	30.0	1280	0.57
efficientnetv2_B1	10.7	1280	0.34
VGG19	204.0	512	0.89

efficientnetV2_B0	7.6	1280	0.29
EfficienNetV2_M	56.4	1280	0.75
VGG16	160.5	512	0.97
MobilenNet	6.0	1024	0.30
MobileNetV2	3.2	1280	0.30

S.14. Dataset

The dataset produced in this work and the tables with the features extracted from images were included in the repositories Github (https://github.com/praoiticica/COVID-plasmonic-sensor-ML) and in Mendeley Data, V1, doi: 10.17632/z4js67w5vc.1.

References

(1) Muri, H.; Hjelme, D. LSPR Coupling and Distribution of Interparticle Distances between Nanoparticles in Hydrogel on Optical Fiber End Face. *Sensors* **2017**, *17* (12), 2723. https://doi.org/10.3390/s17122723.

(2) Minghim, R.; Paulovich, F. V.; de Andrade Lopes, A. Content-Based Text Mapping Using Multi-Dimensional Projections for Exploration of Document Collections. *Vis. Data Anal. 2006* **2006**, *6060*, 60600S. https://doi.org/10.1117/12.650880.

(3) Paulovich, F. V.; Moraes, M. L.; Maki, R. M.; Ferreira, M.; Oliveira, O. N.; De Oliveira, M. C. F. Information Visualization Techniques for Sensing and Biosensing. *Analyst* **2011**, *136* (7), 1344–1350. https://doi.org/10.1039/c0an00822b.

(4) Faloutsos, C.; Lin, K. I. D. FastMap: A Fast Algotithm for Indexing, Data-Mining and Visualization of Traditional and Multimedia Datasets. *ACM SIGMOD Rec.* **1995**, *24* (2), 163–174. https://doi.org/10.1145/568271.223812.

(5) Tejada, E.; Minghim, R.; Nonato, L. G. On Improved Projection Techniques to Support Visual Exploration of Multi-Dimensional Data Sets. *Inf. Vis.* **2003**, *2* (4), 218–231. https://doi.org/10.1057/palgrave.ivs.9500054.

(6) Soares, J. C.; Soares, A. C.; Rodrigues, V. C.; Oiticica, P. R. A.; Raymundo-Pereira, P. A.; Bott-Neto, J. L.; Buscaglia, L. A.; de Castro, L. D. C.; Ribas, L. C.; Scabini, L.; Brazaca, L. C.; Correa, D. S.; Mattoso, L. H. C.; de Oliveira, M. C. F.; de Carvalho, A. C. P. L. F.; Carrilho, E.; Bruno, O. M.; Melendez, M. E.; Oliveira, O. N. Detection of a SARS-CoV-2 Sequence with Genosensors Using Data Analysis Based on Information Visualization and Machine Learning Techniques. *Mater. Chem. Front.* **2021**, *5* (15), 5658–5670. https://doi.org/10.1039/D1QM00665G.

(7) Shimizu, F. M.; de Barros, A.; Braunger, M. L.; Gaal, G.; Riul Jr, A.

Information Visualization and Machine Learning Driven Methods for Impedimetric Biosensing. *TrAC Trends Anal. Chem.* **2023**, *165*, 117115. https://doi.org/10.1016/j.trac.2023.117115.

(8) Oliveira, O. N.; Pavinatto, F. J.; Constantino, C. J. L.; Paulovich, F. V.; de Oliveira, M. C. F. Information Visualization to Enhance Sensitivity and Selectivity in Biosensing. *Biointerphases* **2012**, *7* (1), 53. https://doi.org/10.1007/S13758-012-0053-7.

(9) Haralick, R. M.; Dinstein, I.; Shanmugam, K. Textural Features for Image Classification. *IEEE Trans. Syst. Man Cybern.* **1973**, *SMC-3* (6), 610–621. https://doi.org/10.1109/TSMC.1973.4309314.

(10) Weszka, J. S.; Dyer, C. R.; Rosenfeld, A. A Comparative Study of Texture Measures for Terrain Classification. *IEEE Trans. Syst. Man Cybern.* **1976**, *SMC-6* (4), 269–285. https://doi.org/10.1109/TSMC.1976.5408777.

(11) Ojala, T.; Pietikainen, M.; Maenpaa, T. Multiresolution Gray-Scale and Rotation Invariant Texture Classification with Local Binary Patterns. *IEEE Trans. Pattern Anal. Mach. Intell.* **2002**, *24* (7), 971–987. https://doi.org/10.1109/TPAMI.2002.1017623.

(12) Guo, Z.; Zhang, L.; Zhang, D. A Completed Modeling of Local Binary Pattern Operator for Texture Classification. *IEEE Trans. Image Process.* **2010**, *19* (6), 1657–1663. https://doi.org/10.1109/TIP.2010.2044957.

(13) Shu, X.; Song, Z.; Shi, J.; Huang, S.; Wu, X.-J. Multiple Channels Local Binary Pattern for Color Texture Representation and Classification. *Signal Process. image Commun.* **2021**, *98*, 116392. https://doi.org/10.1016/j.image.2021.116392.

(14) van der Walt, S.; Schönberger, J. L.; Nunez-Iglesias, J.; Boulogne, F.; Warner, J. D.; Yager, N.; Gouillart, E.; Yu, T. Scikit-Image: Image Processing in Python. *PeerJ* **2014**, *2* (1), e453. https://doi.org/10.7717/peerj.453.

(15) Deng, J.; Dong, W.; Socher, R.; Li, L.-J.; Kai Li; Li Fei-Fei. ImageNet: A Large-Scale Hierarchical Image Database. In *2009 IEEE Conference on Computer Vision and Pattern Recognition*; IEEE: Miami, FL, USA, 2009; pp 248–255. https://doi.org/10.1109/CVPR.2009.5206848.

(16) Simonyan, K.; Zisserman, A. Very Deep Convolutional Networks for Large-Scale Image Recognition. *arXiv Prepr.* **2014**. https://doi.org/10.48550/arXiv.1409.1556.

(17) He, K.; Zhang, X.; Ren, S.; Sun, J. Deep Residual Learning for Image Recognition. In *2016 IEEE Conference on Computer Vision and Pattern Recognition (CVPR)*; IEEE, 2016; Vol. 2016-Decem, pp 770–778. https://doi.org/10.1109/CVPR.2016.90.

(18) Huang, G.; Liu, Z.; Van Der Maaten, L.; Weinberger, K. Q. Densely Connected Convolutional Networks. In *2017 IEEE Conference on Computer Vision and Pattern Recognition (CVPR)*; IEEE: Honolulu, HI, USA, 2017; pp 2261–2269. https://doi.org/10.1109/CVPR.2017.243.

(19) Howard, A. G.; Zhu, M.; Chen, B.; Kalenichenko, D.; Wang, W.; Weyand, T.; Andreetto, M.; Adam, H. MobileNets: Efficient Convolutional Neural Networks for Mobile Vision Applications. **2017**.

(20) Sandler, M.; Howard, A.; Zhu, M.; Zhmoginov, A.; Chen, L.-C. MobileNetV2: Inverted Residuals and Linear Bottlenecks. In *2018 IEEE/CVF Conference on Computer Vision and Pattern Recognition*; IEEE: Salt Lake City, UT, USA, 2018; pp 4510–4520. https://doi.org/10.1109/CVPR.2018.00474.

(21) Howard, A.; Sandler, M.; Chen, B.; Wang, W.; Chen, L. C.; Tan, M.; Chu, G.; Vasudevan, V.; Zhu, Y.; Pang, R.; Le, Q.; Adam, H. Searching for MobileNetV3. *Proc. IEEE Int. Conf. Comput. Vis.* **2019**, *2019-October*, 1314–1324. https://doi.org/10.1109/ICCV.2019.00140.

(22) Tan, M.; Le, Q. V. EfficientNetV2: Smaller Models and Faster Training. *arXiv Prepr.* **2021**, *139*, 10096–10106.

(23) Hubert, M.; Van Driessen, K. Fast and Robust Discriminant Analysis. *Comput. Stat. Data Anal.* **2004**, *45* (2), 301–320. https://doi.org/10.1016/S0167-9473(02)00299-2.

(24) James, G.; Witten, D.; Hastie, T.; Tibshirani, R.; Taylor, J. *An Introduction to Statistical Learning*; Springer Texts in Statistics; Springer International Publishing: Cham, 2023. https://doi.org/10.1007/978-3-031-38747-0.

(25) Li, S.; Zhang, H.; Ma, R.; Zhou, J.; Wen, J.; Zhang, B. Linear Discriminant Analysis with Generalized Kernel Constraint for Robust Image Classification. *Pattern Recognit.* **2023**, *136*, 109196. https://doi.org/10.1016/J.PATCOG.2022.109196.

(26) Vapnik, V. N. *The Nature of Statistical Learning Theory*; Springer New York: New York, NY, 2000. https://doi.org/10.1007/978-1-4757-3264-1.

(27) Boser, B. E.; Guyon, I. M.; Vapnik, V. N. A Training Algorithm for Optimal Margin Classifiers. In *Proceedings of the fifth annual workshop on Computational learning theory*; ACM: New York, NY, USA, 1992; pp 144–152. https://doi.org/10.1145/130385.130401.

(28) Cortes, C.; Vapnik, V. Support-Vector Networks. *Mach. Learn.* **1995**, *20* (3), 273–297. https://doi.org/10.1007/BF00994018.

(29) Breiman, L. Random Forests. *Mach. Learn.* **2001**, *45* (1), 5–32. https://doi.org/10.1023/A:1010933404324.

(30) Google. *Welcome to Colaboratory*. https://colab.research.google.com/notebooks/intro.ipynb (accessed 2024-04-02).
